# Enhancing genomic prediction with Stacking Ensemble Learning in Arabica Coffee

**DOI:** 10.3389/fpls.2024.1373318

**Published:** 2024-07-17

**Authors:** Moyses Nascimento, Ana Carolina Campana Nascimento, Camila Ferreira Azevedo, Antonio Carlos Baiao de Oliveira, Eveline Teixeira Caixeta, Diego Jarquin

**Affiliations:** ^1^ Laboratory of Intelligence Computational and Statistical Learning (LICAE), Department of Statistics, Federal University of Viçosa, Viçosa, Brazil; ^2^ Agronomy Department, University of Florida, Gainesville, FL, United States; ^3^ Embrapa Coffee, Brazilian Agricultural Research Corporation (Embrapa), Brasília, Brazil

**Keywords:** statistical and machine learning, prediction accuracy, plant breeding, ensemble methods, GBLUP

## Abstract

Coffee Breeding programs have traditionally relied on observing plant characteristics over years, a slow and costly process. Genomic selection (GS) offers a DNA-based alternative for faster selection of superior cultivars. Stacking Ensemble Learning (SEL) combines multiple models for potentially even more accurate selection. This study explores SEL potential in coffee breeding, aiming to improve prediction accuracy for important traits [yield (YL), total number of the fruits (NF), leaf miner infestation (LM), and cercosporiosis incidence (Cer)] in Coffea Arabica. We analyzed data from 195 individuals genotyped for 21,211 single-nucleotide polymorphism (SNP) markers. To comprehensively assess model performance, we employed a cross-validation (CV) scheme. Genomic Best Linear Unbiased Prediction (GBLUP), multivariate adaptive regression splines (MARS), Quantile Random Forest (QRF), and Random Forest (RF) served as base learners. For the meta-learner within the SEL framework, various options were explored, including Ridge Regression, RF, GBLUP, and Single Average. The SEL method was able to predict the predictive ability (PA) of important traits in Coffea Arabica. SEL presented higher PA compared with those obtained for all base learner methods. The gains in PA in relation to GBLUP were 87.44% (the ratio between the PA obtained from best Stacking model and the GBLUP), 37.83%, 199.82%, and 14.59% for YL, NF, LM and Cer, respectively. Overall, SEL presents a promising approach for GS. By combining predictions from multiple models, SEL can potentially enhance the PA of GS for complex traits.

## 1 Introduction

Coffee is one of the most globally beverages presenting importance in terms of its potential health, socioeconomic, and economic effects (Porto et al., 2019). These effects drive breeding programs worldwide to develop high-yielding, adaptable cultivars delivering superior bean quality (Barbosa et al., 2019). However, traditional selection methods based on phenotypic observations of the plants or their family history (pedigree) are expensive and time consuming, especially for perennial crops as coffee.

An alternative approach denoted genomic selection (GS) has been used as a successful tool in genetic improvement (Meuwissen et al., 2001). GS helps increase genetic gain per generation by allowing for earlier selection through improved prediction of the potential of individual plants (Daetwyler et al., 2013; Nascimento et al., 2019; Voss-Fels et al., 2019). In the field of coffee breeding, GS has been utilized with the dual aim of accelerating genetic gain through early selection and improving prediction accuracy (Sousa et al., 2019; Alkimim et al., 2020; Sousa et al., 2021; Coelho de Sousa et al., 2022).

Among several prediction models, Genomic Best Linear Unbiased Prediction (GBLUP) is the most widely used approach for genomic prediction due to its advantages (Zhang et al., 2021). Compared to other parametric methods, GBLUP allows to accurately estimate narrow-sense heritability (Li et al., 2019) and presents higher computational efficiency (Hernandez et al., 2020). GBLUP modeling is also flexible. It can be modified to incorporate additional genetic information beyond the typical single-nucleotide polymorphism (SNP) markers. Specifically, this modeling allows to account for non-additive genetic effects, environmental factors, and even genotype-by-environment interactions, enriching the analysis and potentially improving prediction accuracy (Jarquín et al., 2014).

In the Artificial Intelligence Era, the interest in semi- and non-parametric methods for GS is increasing (Larkin et al., 2019; Coelho de Sousa et al., 2022; Seyum et al., 2022). These approaches, such as Artificial Neural Networks and Decision Trees, do not require prior assumptions about the relationships between inputs (SNP markers) and the output (phenotypic observations), allowing great flexibility to handle complex non-additive effects, such as dominance and epistasis (McKinney et al., 2006; Abdollahi-Arpanahi et al., 2020; Coelho de Sousa et al., 2022). In general, despite their potential, these approaches do not outperform the traditional parametric methods (e.g., GBLUP, Bayesian Alphabet - Gianola et al., 2009) used to predict the genetic merit of individuals (Liang et al., 2021).

Aiming to enhance predictive ability (PA), Ensemble Learning (EL) combines predictions from multiple models (base learners) into a single prediction (meta-learner) (Mendes-Moreira et al., 2012; Ganaie et al., 2022; Mienye and Sun, 2022). This approach leverages the strengths of diverse models to potentially generate more robust results compared to relying on a single learner (Liang et al., 2021; Kalule et al., 2023). In the context of GS, EL has found application through methods such as Random Forest (RF) and Bagging (Bag) (Xu et al., 2019; Abdollahi-Arpanahi et al., 2020; Sousa et al., 2021; Costa et al., 2022). These methods, categorized as Homogenous Learning (HL), utilize a single framework to produce a single prediction value. Conversely, the Stacking Ensembles Learning (SEL) approach combines predictions from diverse methods, potentially outperforming HL (Mendes-Moreira et al., 2012). SEL has seen success in GS, improving PA in Chinese Simmental cattle, Dutch cattle, and pine (Liang et al., 2021), achieving higher accuracy than GBLUP for most evaluated traits.

Despite being interesting, EL approach arises some issues that needs to be considered. First, since the same individuals are used to fitting the model(s) in the EL approach, it is expected the existence of correlation between the predictions derived from the different methods. This well-known statistical problem is referred as multicollinearity (Montgomery et al., 2021) and causes high variability of the estimated effects. The second issue is related to which dataset should be used to fit the meta-learner. One option is to use directly the predicted values derived from the base learners. In this case, the simple mean or some regression model that accounts for multicollinearity problem (e.g., Ridge Regression) can be implemented to makeup a single prediction. An alternative option also could consider combining the predicted values with the genomic covariates (i.e., SNP markers|predicted values from the base learners) with the previous training data as new inputs. In this regard, in addition to the multicollinearity, the course of the dimensionality is another issue to consider mainly because these are covariables of different type.

Liang et al. (2021) used the predicted values derived from a multiple regression model as meta-learner. These authors obtained good results to improve PA compared to the conventional genomic prediction models on three different datasets. However, the use of an expanded training data augmented by SNP markers could be beneficial to further enhance the PA, and it emerges as an interesting approach. In this case, a model that addresses both multicollinearity and dimensionality problems should be used. One of the possible solutions can be considered to use a two-kernel GBLUP model as the meta-learner model. Another approach to evaluate is to consider only the predicted values provided by the best base learner models.

To date, no research has applied SEL to improve the prediction accuracy of important traits in coffee cultivars. This approach presents potential for coffee breeding, as it has been shown to outperform standard methods in other applications (Liang et al., 2021; Mohammed and Kora, 2023). By combining the strengths of multiple prediction models, SEL could lead to more reliable and accurate identification of valuable genetic traits in coffee plants, accelerating the development of superior coffee varieties.

In light of the mentioned points, the objective of this study was to use and evaluate the SEL to improve PA of important traits in Coffea Arabica. For that, the GBLUP, multivariate adaptive regression splines (MARS), Quantile Random Forest (QRF), and RF models were used as the base learner. Several approaches were considered as the meta-learner to construct the SEL framework. Specifically, the expanded- and non-expanded datasets were used for training. In addition, models that account for multicollinearity (Ridge Regression) and multicollinearity and dimensionality jointly (GBLUP) were also implemented.

## 2 Materials and methods

### 2.1 Phenotypic and genotypic data

The data were collected from the C. arabica breeding program, which is a joint partnership among the Agricultural Research Company of Minas Gerais (EPAMIG), the Federal University of Viçosa (UFV), and the Brazilian Agricultural Research Corporation (EMBRAPA). An experimental area is maintained at the Department of Phytopathology—UFV (20°44′25” S, 42°50′52” W). The database is composed of 13 progenies derived from crosses between three parents of the Catuaí cultivar and three parents of the Híbrido de Timor (HdT). Fifteen genotypes of the abovementioned progeny set (totaling 195 individuals) were genotyped for 21,211 SNP markers by Rapid Genomics, located in Gainesville Florida, USA. Information about the probes design and SNP identification are detailed in Sousa et al. (2017). The SNP markers set are widely distributed in the genome and in all coffee chromosomes, being useful for accurate studies on diversity and population structure, as well as selection and genomic association in C. arabica (Sousa et al., 2017, Sousa et al., 2019). The SNP quality control was carried out considering genotypic call rate and minor allele frequency equal to or greater than 90% and smaller than 5%, respectively. In this study a pre-selected set of 5,970 markers that did not reduce the PA of Arabica Coffee compared to the original set SNP markers in a previous study was used (Arcanjo et al., 2024).

The genotypes were planted on February 11, 2011, using a spacing of 3.0 m between rows and 0.7 m between plants following an augmented (check varieties) blocks experimental design. Nutritional management was carried out following the requirements of the crop. The phenotypic evaluations were performed in 2014, 2015, and 2016. A total of four traits were scored, two associated with the productivity, yield (YL—liters of fresh cherries harvested per plant) and total number of fruits (NF) —and two more associated with disease resistance—leaf miner infestation (LM) and cercosporiosis incidence (Cer) in Coffea Arabica. The incidence of cercosporiosis and leaf miner was evaluated using a score scale ranging from 1 to 5, in which 1 corresponded to genotypes without symptoms and 5 referred to highly susceptible genotypes. A comprehensive description of how the evaluations of each trait were performed can be found in Sousa et al. (2019).

### 2.2 Phenotypic data analysis

The phenotypic data for YL, NF, LM, and Cer were analyzed according to the following statistical model


y=Xu+Zg+Wp+Vr+Tb+Ri+e

where y represents the vector of observed phenotypes; u is the vector referring to the general mean in each evaluation year; g is the vector of genetic random effects corresponding to the progeny such that $g\;∼\text{N}\left(0,\text{I}{\text{σ}}_{g}^{2}\right)$ ; p is the random permanent environmental effect $p\;∼\text{N}\left(0,\text{I}{\text{σ}}_{p}^{2}\right)$ ; r is the population random effect $r\;∼\text{N}\left(0,\text{I}{\text{σ}}_{r}^{2}\right)$ ; b is the plot random effect $b\;∼\text{N}\left(0,\text{I}{\text{σ}}_{b}^{2}\right)$ ; **i** corresponds to the random effect of the interaction between progenies and the years $i\;∼\text{N}\left(0,\text{I}{\text{σ}}_{i}^{2}\right)$ ; and **e** is the experimental error assumed to be Independent and Identically Distributed (IID) outcomes from a normal density such that $\;e\;∼\text{N}\left(0,\text{I}{\text{σ}}_{e}^{2}\right)$ . The genetic parameters, heritability and correlation, were also estimated for the evaluated traits. The individual heritability was estimated by $h^{2}=\frac{{\text{σ}}_{g}^{2}}{{\text{σ}}_{g}^{2}+{\text{σ}}_{p}^{2}+{\text{σ}}_{r}^{2}+{\text{σ}}_{i}^{2}+{\text{σ}}_{e}^{2}}\;$ . In addition, the adjusted phenotypes (y*, corrected BLUPs) for the year, plot, and year × progenies interaction effects were calculated and used for GS. The analyses were carried out using Selegen-REML/BLUP *software* (de Resende, 2016).

### 2.3 Individual genomic prediction

#### 2.3.1 GBLUP

The parameterization of the Genomic prediction G-BLUP model can be defined as follows


$$y^{*}=Xb+Zu+e$$

where $y^{*}$ is the vector of adjusted phenotypic observations as previously detailed; b  is the vector of means; **X** is the incidence matrix corresponding to the fixed effects; u  is the vector of individual additive genomic effects such that $u∼\text{N}\left(0,G{\text{σ}}_{g}^{2}\right)\;$ where G  is the kinship matrix describing genomic similarities between pairs of individuals, ${\text{σ}}_{g}^{2}$ is the additive genetic variance, **Z** is the incidence matrix that connect phenotypes with genotypes; e  is the random error vector with $e∼\text{N}\left(0,I{\text{σ}}_{e}^{2}\right)\;$ where ${\text{σ}}_{e}^{2}$ is the residual variance. The additive genomic kinship matrix **G** was obtained as described by VanRaden (2008)


$$G=\frac{W^{T}W}{\sum_{\text{i}=1}^{\text{n}}2{\text{p}}_{\text{i}}\left(1-{\text{p}}_{\text{i}}\right)}$$

where, W  is the centered (by columns) matrix of SNPs, which specifies the marker genotypes for each individual as 0, 1 or 2; ${\text{p}}_{\text{i}}\;$ is the frequency of the second allele at the locus, that is,


$${\text{W}}_{\text{ij}}=\{\begin{array}{c} 2-2{\text{p}}_{\text{j }},\;\;\text{if}\;{\text{M}}_{\text{ij}}=\text{AA}\;\\ 1-2{\text{p}}_{\text{j}},\;\;\text{if}\;\;{\text{M}}_{\text{ij}}=\text{Aa}\\ -2{\text{p}}_{\text{j}},\;\text{if}\;\;{\text{M}}_{\text{ij}}=\text{aa} \end{array}$$

The *BGLR* function of the BGLR package (Pérez and de los Campos, 2014) in R software (R Core Team, 2022) was used to fitting GBLUP model.

#### 2.3.2 Decision tree

The decision tree structure in this case is built using a regression tree algorithm. The objective is to create regions (R_1_, R_2_,…, R_M_) that minimize the difference between the predicted values and the adjusted observed values. This difference is measured by the Residual Sum of Squares (RSS). To achieve this, the algorithm performs a recursive binary splitting process. At each step, it considers all available features ( $X_{j}-\;$ markers) and all possible split points (cutoff values) within each feature. The split that results in the lowest RSS for the resulting child nodes is chosen. This process continues recursively until a stopping criterion is met, such as reaching a minimum number of data points in a region. Mathematically, the two disjoint regions can be defined by (Hastie et al., 2009)


$${\text{R}}_{1}\left(\text{j},\text{s}\right)=\left\{\text{X}|{\text{X}}_{\text{j}}<\text{s}\right\}\text{ and }{\text{R}}_{2}\left(\text{j},\text{s}\right)=\left\{\text{X}|{\text{X}}_{\text{j}}\geq \text{s}\right\},$$

and the goal is to minimize:


$$\sum_{\text{i}:{\text{x}}_{\text{i}}\in {\text{R}}_{1}\left(\text{j},\text{s}\right)}{\left({\text{y}}_{i}^{*}-{\hat{y}}_{{\text{R}}_{1}}^{*}\right)}^{2}+\sum_{\text{i}:{\text{x}}_{\text{i}}\in {\text{R}}_{2}\left(\text{j},\text{s}\right)}{\left({\text{y}}_{i}^{*}-{\hat{y}}_{{\text{R}}_{2}}^{*}\right)}^{2}$$

where ${\hat{y}}_{{\text{R}}_{1}}^{*}\;$ is the average of the adjusted phenotypic values of the training observations belonging to the region ${\text{R}}_{1}\left(\text{j},\text{ s}\right)=\;\left\{{\text{X|X}}_{\text{j}}\;<\text{ s}\right\}$ , ${\hat{y}}_{{\text{R}}_{2}}^{*}$ is the average of the adjusted phenotypic values of the training observations belonging to the region ${\text{R}}_{2}\left(\text{j},\text{ s}\right)=\;\left\{{\text{X|X}}_{\text{j}}\;\geq \text{ s}\right\}\text{ and }{\text{y}}_{i}^{*}$ is the true value of each individual.

#### 2.3.3 Random Forest

To construct a RF is necessary to create several datasets by resampling (bootstrapping) from the training set. After that, the bootstrap samples are used to build multiple trees considering a subset of predictors (markers) randomly selected (Hastie et al., 2009). Usually, for a continuous response, the number of predictors used to find the best split at each node is a subset that is chosen by $m=\;\frac{v}{3}$ , with v being the total number of predictors. Also, usually, the number of trees for the RF is set to 500. For the RF, the trees grow to their maximum size without pruning, and the prediction is done by averaging the trees. The function *randomForest* in randomForest R-package (Liaw and Wiener, 2002) was used to implement RF method.

#### 2.3.4 Quantile Random Forest

For the construction of the QRF, as same as for RF, it is necessary to obtain T regression trees generated from bootstrap samples considering subsets of the markers under study (Hastie et al., 2009). Then, for the *t* ^th^ generated tree ( ${\text{T}}_{\text{t}}$ ), the conditional distribution is obtained by weighting the observed values of the studied traits. Specifically, given an observation, X =x , it is defined for each terminal node (adjusted tree leaf), $\text{F}\left(\text{x},{\text{T}}_{\text{tf}}\;\right)$ , the following weighting factor: ${\text{w}}_{\text{i}}\left(\text{x},{\text{T}}_{\text{tf}}\;\right)=\;\frac{{\text{I}}_{\left\{\text{x}\in \text{F}\left(\text{x},{\text{T}}_{\text{tf}}\;\right)\;\right\}}}{\#\left\{\text{m}:{\text{ X}}_{\text{m}}\in \text{F}\left(\text{x},{\text{T}}_{\text{tf}}\;\right)\right\}},$ with $\sum_{\text{i}=1}^{\text{n}}{\text{w}}_{\text{i}}\left(\text{x},{\text{T}}_{\text{tf}}\;\right)=1$ , ${\text{I}}_{\left\{{\text{X}}_{\text{i}}\in \text{F}\left({\text{x}}_{\text{i}},{\text{T}}_{\text{tf}}\;\right)\;\right\}}$ an indicator variable stating that the observed value ( X =x ) belongs to *f* ^th^ leaf and $\#\left\{\text{m}:{\text{ X}}_{\text{m}}\in \text{F}\left(\text{x},{\text{T}}_{\text{tf}}\;\right)\right\}$ represents the number of observations on the *f* ^th^ leaf.

The prediction of a tree ${\text{T}}_{\text{t}}$ , according to Meinshausen (2006), for a new point, $\text{X }={\text{x}}_{\text{new}},$ is given by the weighted average of the observations ${\text{Y}}_{\text{i}}$ , that is, $\hat{\text{μ}}\left({\text{x}}_{\text{new}}\right)=\sum_{\text{i}=1}^{\text{n}}{\text{w}}_{\text{i}}\left(\text{x},{\text{T}}_{\text{tf}}\;\right){\text{Y}}_{\text{i}}$ . In this way, the prediction for a given observation, X =x, after the construction of T trees is given by ${\hat{\text{μ}}}_{\text{RF}}\left(\text{x}\right)=\sum_{\text{i}=1}^{\text{n}}{\text{w}}_{\text{i}}\left(\text{x}\right){\text{Y}}_{\text{i}}$ where ${\text{w}}_{\text{i}}\left(\text{x}\right)=\frac{1}{\text{T}}\sum_{\text{t}=1}^{\text{T}}{\text{w}}_{\text{i}}\;(\text{x},{\text{T}}_{\text{tf}}\;)$ . Taking into consideration that the estimated cumulative distribution function is given by $\hat{\text{F}}\left(\text{y}|\text{X}=\text{x}\right)=\sum_{\text{i}=1}^{\text{n}}{\text{w}}_{\text{i}}\left(\text{x}\right){\text{I}}_{\left\{{\text{Y}}_{\text{i}}\leq \text{y}\right\}}$ , where ${\text{I}}_{\left\{{\text{Y}}_{\text{i}}\leq \text{y}\right\}}$ is an indicator function, the predicted value for the ${\text{τ}}^{th}$ quantile is given by ${\text{Q}}_{\text{τ}}(\text{x})=\inf\;\{\text{y}:\;\hat{\text{F}}(\text{y}|\text{X}=\text{x})\;\geq \text{ τ }\}$ , for any τ,  0<τ<1  .

The main difference between QRF and RF is that, for each node in each tree, the RF maintains only the average of the observations that fall into that node and discards any other information. Conversely, the QRF maintains the value of all node observations (not just the average) and evaluates the conditional distribution based on this information (Meinshausen, 2006). This study evaluated nine Quantile Random Forest (QRF) for various quantile levels. The quantile parameter (τ) ranged from 0.1 to 0.9 in increments of 0.1. Therefore, the models were named QRF0.1, QRF0.2,…, QRF0.9, reflecting the specific quantile they aimed to predict. The function *quantregForest* in quantregForest R-package (Meinshausen, 2017) was used to implement the QRF methods.

#### 2.3.5 Multivariate adaptive regression splines

MARS (Friedman, 1991) forms reflexive pairs of base functions (BF) for each input (marker) ${\text{X}}_{\text{j}}$ , with nodes at each observed value ${\text{x}}_{\text{ij}}$ of that input. The model building strategy is like a progressive linear regression, but instead of using the original inputs, it implements base functions from the set $\text{C}={\left\{{\left({\text{X}}_{\text{j}}-\text{t}\right)}_{+},\;{\left(\text{t}-{\text{X}}_{\text{j}}\right)}_{+}\right\}}_{\text{ t }\in \left\{{\text{x}}_{1\text{j}},{\text{ x}}_{2\text{j}},\;\ldots ,{\text{ x}}_{\text{Nj}}\right\}\text{ j}=1,2,\ldots ,\text{ p }}$ and/or its products. The MARS model, which is a linear combination of the BF and/or their interactions, is given by (Hastie et al., 2009):


$$\text{f}\left(\text{X}\right)={\text{ β}}_{0}+\sum_{m=1}^{M}{\text{β}}_{\text{m}}{\text{h}}_{\text{m}}\left(\text{X}\right)$$

where ${\text{β}}_{0}$ is the regression constant, ${\text{β}}_{\text{m}}$ with *m* = 1, 2,…, *M*, are the regression coefficients, and ${\text{h}}_{m}\left(\text{X}\right)$ is a function in C , or a product of two or more functions.

The estimation process of the parameters $\beta_{0}$ and $\beta_{m}$ is based on the minimization of the residual sum of squares. First, the forward phase starts on the training data, building the model initially with only the constant function $h_{0}\left(X\right)=1$ , and all functions in the C set are candidate functions. At each subsequent step, the base pair that produces the maximum reduction in training error is added. Considering a model with basic M functions, the next pair to be added to the model is


$${\hat{\text{β}}}_{\text{M}+1}{\text{h}}_{\text{l}}\left(\text{X}\right){\left({\text{X}}_{\text{j}}-\text{t}\right)}_{+}+{\hat{\text{β}}}_{\text{M}+2}{\text{h}}_{\text{l}}\left(\text{X}\right){\left(\text{t}-{\text{X}}_{\text{j}}\right)}_{+},{\text{ h}}_{\text{l}}\in \text{M}$$

where ${\hat{\text{β}}}_{\text{M}+1}$ and ${\hat{\text{β}}}_{\text{M}+2}$ are coefficients estimated by the least square method (Hastie et al., 2009), together with all other M+1 coefficients in the model. This process of adding BF continues until the model reaches a predetermined maximum number, often leading to a purposefully overparametrized model (Zhang and Goh, 2016). The backward phase improves the model by removing the least significant terms until finding the best sub model. The model subsets are compared using the generalized cross-validation (GCV) method. The GCV is evaluated with the root-mean-square residual error divided by a penalty that depends on the complexity of the model (Zhang and Goh, 2016) and it is calculated as


$$\text{GCV}\left(\text{λ}\right)=\frac{\frac{1}{\text{N}}\sum_{\text{i}=1}^{\text{N}}{\left[{\text{y}}_{\text{i}}-{\hat{\text{f}}}_{\text{λ}}\left({\text{x}}_{\text{i}}\right)\right]}^{2}}{{\left[1-\frac{\text{C}\left(\text{M}\right)}{\text{N}}\right]}^{2}}$$

(Hastie et al., 2009) where M is the effective number of model parameters, C(M) is a cost function for each basis function included in the developed submodel, which by default adopts the value of 3, N is the number of datasets used in CV and ${\hat{\text{f}}}_{\text{λ}}\left({\text{x}}_{\text{i}}\right)$ denotes the predicted MARS values. This study employed three Adaptive Regression Spline (MARS) models with varying degrees of interaction (1, 2, and 3). The MARS 1 model represents an additive model, meaning it captures only the linear effects of markers. In contrast, MARS 2 and MARS 3 allow for the inclusion of second and third-order interactions, respectively, enabling them to capture more complex non-additive relationships between markers. The function *earth* in *earth* R-package (Milborrow, 2017) was used to implement MARS models.

### 2.4 Stacking Ensemble Learning for genomic prediction

**Figure 1:**
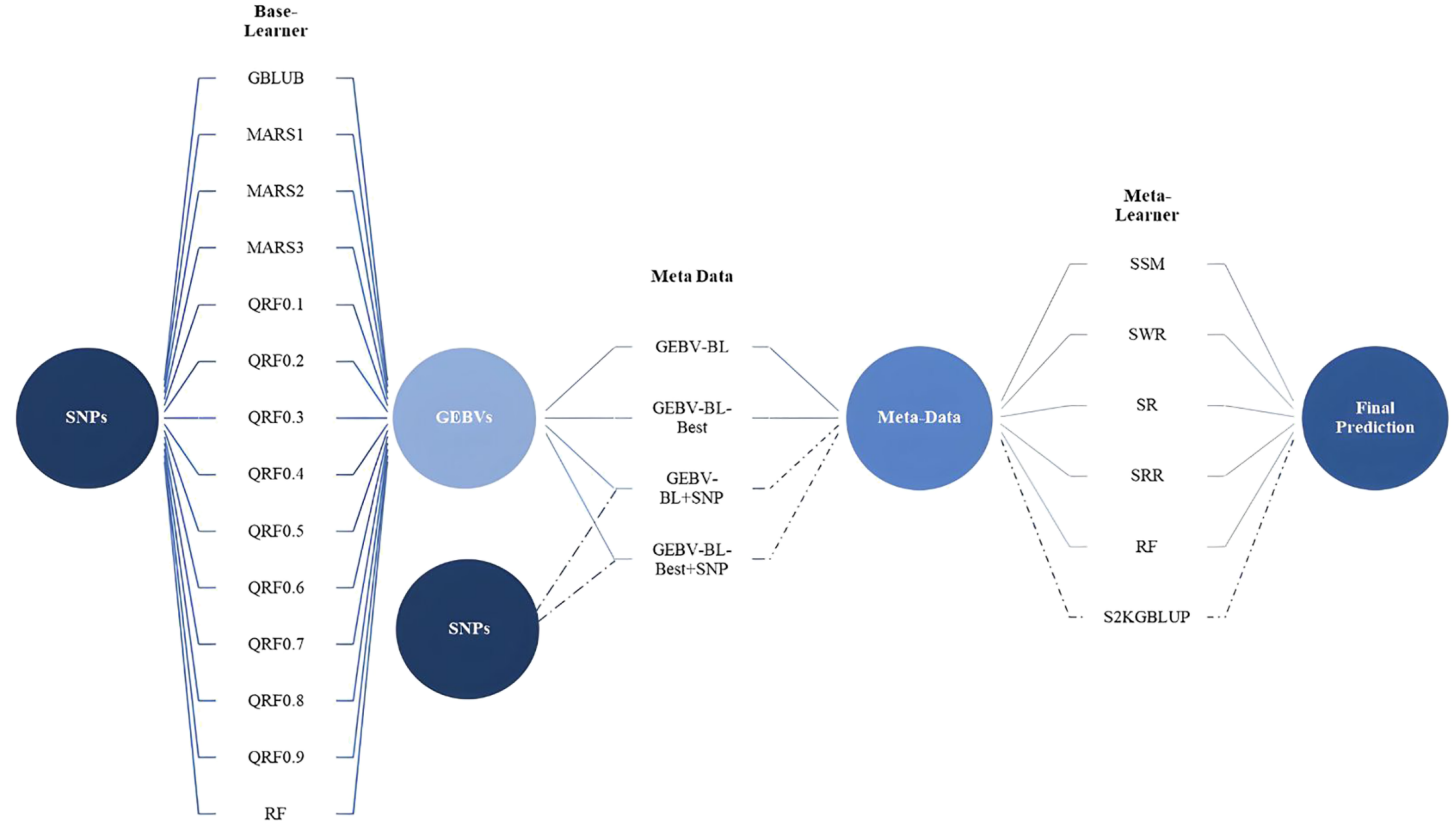
The stacking ensemble learning framework for genomic prediction from original data to the base learners, creating metadata for the meta-learner. Base-Learner (Level 0) is composed of the GBLUP, MARS (1°, 2°, and 3°), QRF considering nine quantiles (from 0.1 to 0.9, in steps of 0.1) and RF model. Four different meta-data were obtained: (i) GEBV-BL, only predictions from the base learners (standard approach); (ii) GEBV-BL+SNP, predictions combined with the original Single Nucleotide Polymorphism (SNP) markers (larger input dataset); (iii) GEBV-BL-Best; and (iv) GEBV-BL-Best + SNP, similar to the previous cases, but only predictions from high-performing base learners (those exceeding the average predictive accuracy). Meta-Learners: for GEBV-BL and GEBV-BL-Best metadata, six meta-learner methods were evaluated. Simple Mean (SSM), Weighted Regression (SWR), Regression (SR), Ridge Regression (SRR), Random Forest (SRF), and for GEBV-BL + SNP and GEBV-BL-Best + SNP datasets, which included SNP markers, a two-kernel GBLUP model (S2KGBLUP).

This study explores the SEL approach for improving the accuracy of genomic prediction models. SEL leverages predictions from multiple individual models (base learners, Level 0) and combines them using a meta-learner model (Level 1) to generate a final, potentially more accurate prediction. The base learners used in this study were GBLUP, different degrees of MARS (1, 2, 3), multiple QRFs (0.1 to 0.9), and RF. Their predictions, referred to as Genomic Estimated Breeding Values from Base Learners (GEBV-BL), formed the core metadata for the meta-learner. In practice, it is necessary to prepare a dataset with both the observable characteristics (phenotype) and the genetic information (SNP markers) of individuals. Then, diverse machine learning models (base learners) are trained on these data to make initial predictions. These predictions from the base learners become the new features for a final model, the meta-learner. Finally, the meta-learner is trained using these base learner predictions as input and the original phenotype data as the target variable. In our work, four different combinations of metadata were explored: (i) GEBV-BL, only predictions from the base learners (standard approach); (ii) GEBV-BL+SNP, predictions combined with the original Single Nucleotide Polymorphism (SNP) markers (larger input dataset); (iii) GEBV-BL-Best; and (iv) GEBV-BL-Best + SNP, Similar to the previous cases, but only predictions from high-performing base learners (those exceeding the average predictive accuracy) were included. For GEBV-BL and GEBV-BL-Best datasets, six meta-learner methods were evaluated: Simple Mean (SSM); Weighted Regression (SWR); Regression (SR); Ridge Regression (SRR); Random Forest (SRF). For GEBV-BL + SNP and GEBV-BL-Best + SNP datasets, which included SNP markers, a two-kernel GBLUP model (S2KGBLUP) was additionally employed as the meta-learner. The SEL scheme for genomic prediction is illustrated in the **Figure 1**.

### 2.5 Cross-validation

The PA of the models used as base-learners and the entire SEL process considered a CV scheme that was implemented as follows. First, the complete dataset under study was randomly divided into two sets (training and testing). The training set was composed by 70% of the individuals while the remaining 30% was assigned to the testing or validation set. The training set was used to calibrate the base-learners and the SEL for predicting the GEBVs of the individuals in the training set. This procedure was repeated 10 times. Then, for each approach, the average PA across replicated was computed. The PA was computed as the Pearson correlation between predicted GEBV and the adjusted phenotype values. The standard error (SE) was also computed. In addition, the mean square error (MSE) between the observed and predicted values was calculated. Finally, the agreement coefficient was used to compute the percentage of individuals with a performance above the 90^th^ percentile in fields given the top 10% of the GEBVs obtained with the different genomic prediction approaches.

## 3 Results

### 3.1 Phenotypic data analysis

The across environments mean ( $\bar{\text{X}})$ and standard deviation (SD) of the evaluated traits are summarized in **Table 1**.

**Table 1:** Across environments phenotypic mean ( $\bar{\text{X}}$ ) and standard deviation (SD) for yield (YL), total number of fruits (NF), leaf miner infestation (LM), and cercosporiosis incidence (Cer) of a coffea arabica L. population composed of 195 individuals observed in years 2014, 2015, and 2016 in Viçosa, Brazil.

Trait	$\bar{X}$	SD
YL	5.16	3.84
NF	2.32	0.55
LM	2.05	0.69
Cer	2.48	0.70

**Figure 2:**
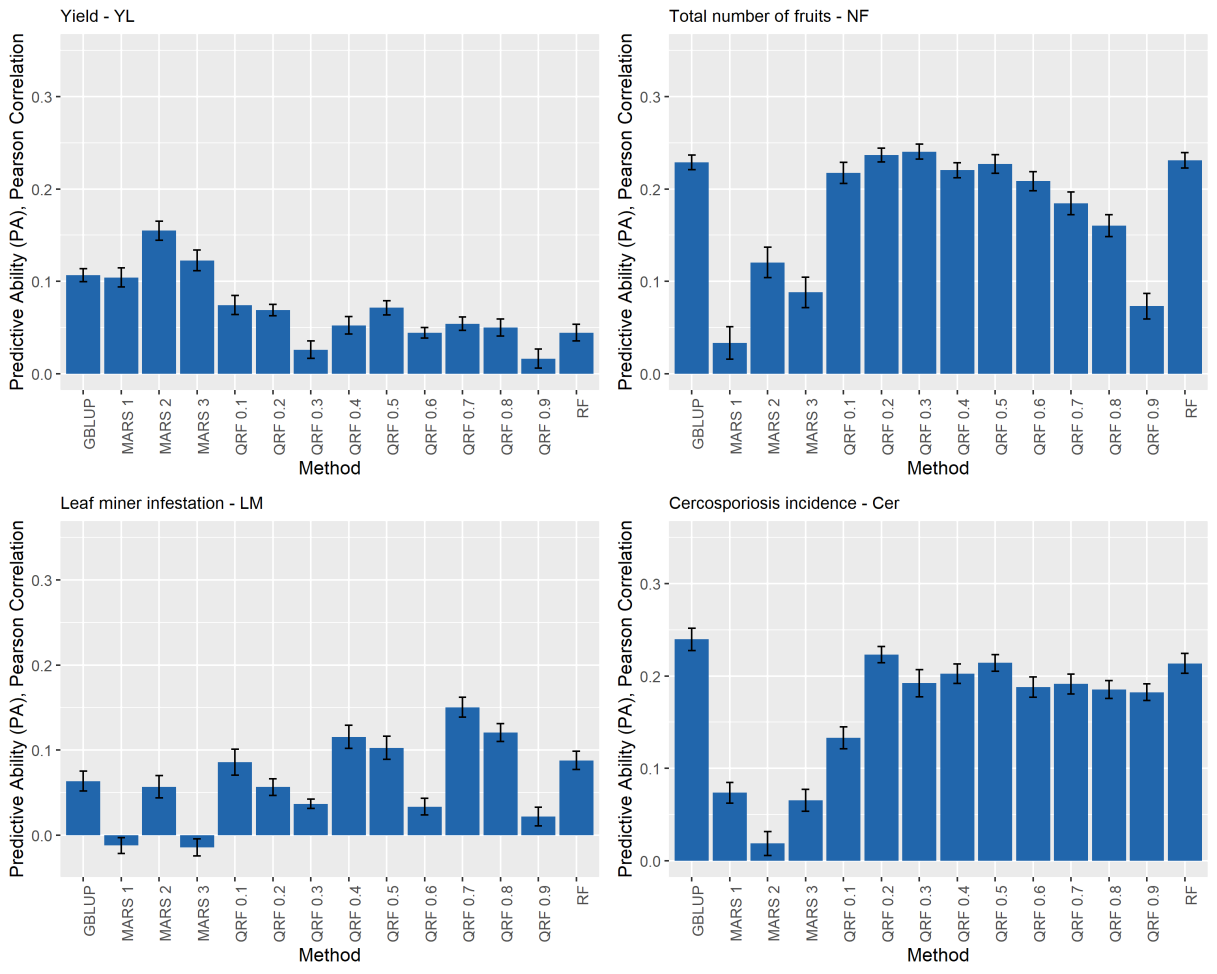
Predictive ability (PA) for yield (YL), total number of fruits (NF), leaf miner infestation (LM), and cercosporiosis incidence (Cer) measured in an Arabica coffee population composed of 195 individuals using a holdout validation scheme repeated 10 times. The fitted models used as base learners are: Genomic Best Linear Unbiased Predictor (GBLUP); Multivariate Adaptive Regression Splines with degrees equal to 1, 2, and 3 (MARS 1, MARS 2 and MARS 3); Quantile Random Forest evaluated at nine quantiles [(τ): 0.1 to 0.9, every 0.1] – (QRF 0.1, …, QRF 0.9), and Random Forest (RF).

The estimates of the heritability (proportion of phenotypic variability explained by the genetic component) for YL (0.30), NF (0.49), LM (0.30), and Cer (0.38) were moderate. The Spearman’s correlation (lower triangle) between the adjusted phenotypic values of each pair of traits were positive and presented low to moderate values varying from 0.02 to 0.52. The higher and the lower correlation values were observed between YL and NF (0.52) and between NF and Cer (0.02, not statistically significant), respectively (**Figure 2**). The correlation between YL and LM, Cer and NF and LM, Cer were not statistically significant (**Supplementary Figure S1**).

### 3.2 Comparison between the base learners

**Figure 3:**
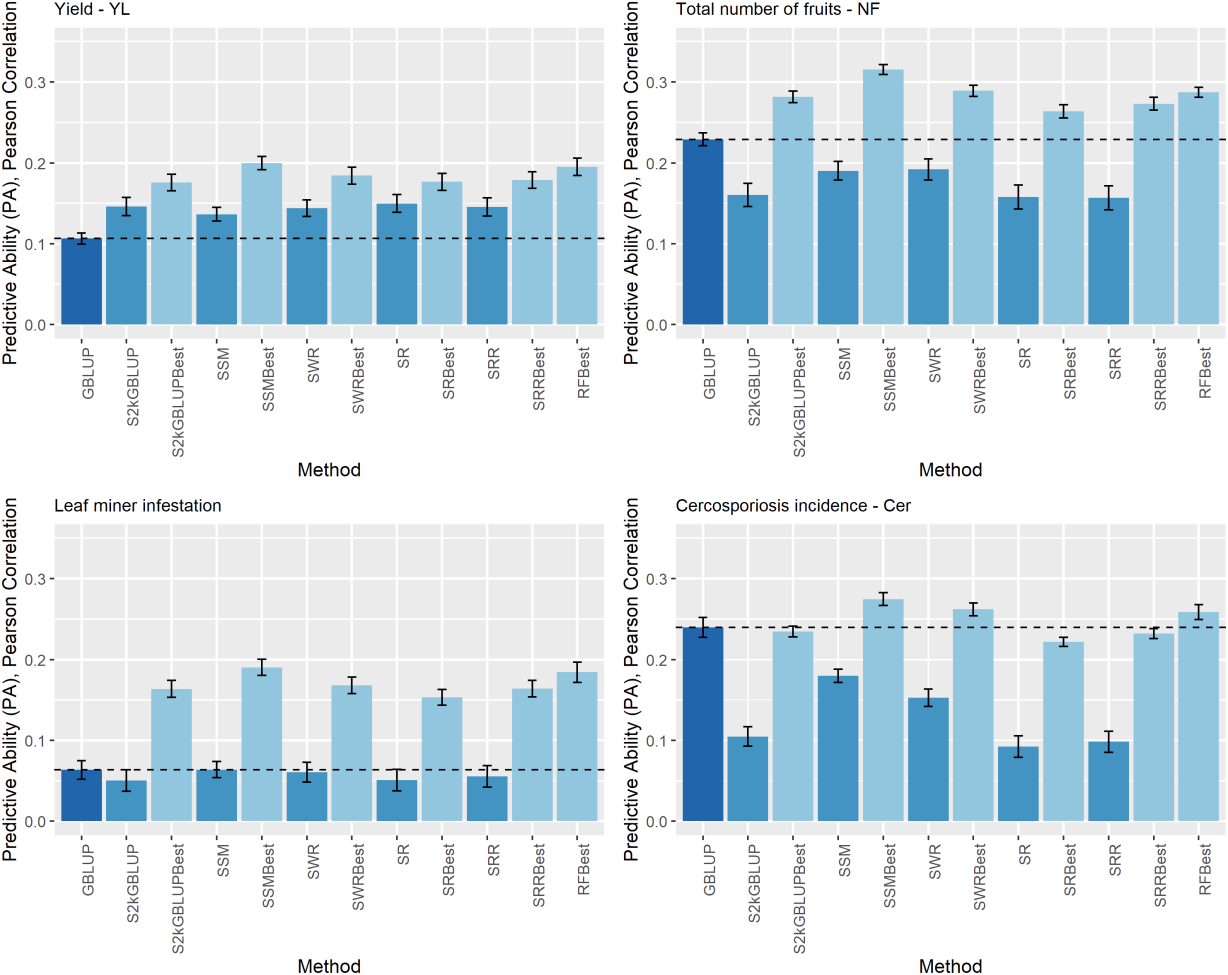
Predictive ability (PA) for yield (YL), total number of fruits (NF), leaf miner infestation (LM), and cercosporiosis incidence (Cer) measured in an Arabica coffee population composed of 195 individuals using a holdout validation scheme repeated 10 times. The fitted models used as base learners are: Stacking Simple Mean (SSM), Stacking Weighed Regression (SWR), Stacking Regression (SR), Stacking Ridge Regression (SRR), and the Stacking two-kernel GBLUP model (S2KGBLUP). The models named as best (SSMBest, SWRBest, SRBest, SRRBest, S2KGBLUP, and RFBest) used in the fitting only the results provided by those methods that presented predictive ability higher the mean in the Level 0.

Overall, none of the evaluated base learner methods outperformed the predictive performance of the others for all the evaluated traits. The estimated predictive abilities (PA) and corresponding standard deviations for the four traits (YL, NF, LM, and Cer) ranged from −0.01 (0.01) to 0.24 (0.01) and are presented in **Figure 3**. Specifically, for YL, NF, LM, and Cer, the highest PA values were 0.15 (0.01), 0.24 (0.01), 0.15 (0.01) and 0.24 (0.02), and these were obtained with MARS2 and QRF0.3, QRF0.7 and GBLUP methods, respectively (**Figure 2**).

GBLUP presented lower values of EQM (**Supplementary Table S1**). Specifically, the MSE were equal to 16.05 (1.01), 2,329.00 (368.94), 0.21 (0.02), and 0.57 (0.07) for YL, NF, LM, and Cer, respectively.

The extreme QRF models QRF0.1, and QRF0.9 returned the highest MSE values across all the evaluated traits (**Supplementary Table S1**). In general, the MSE decreased as the fitted quantile model was approaching to the median model (QRF0.5).

### 3.3 Comparison between the Stacking Ensemble Learning approaches and GBLUP

The estimates of the PA obtained with the SEL models and the traditional genomic prediction method GBLUP model are shown in **Figure 3**. The results of the GBLUP model were used as benchmark since as it was mentioned it is the most convenient and used implementation in genomic prediction.

**Figure 4:**
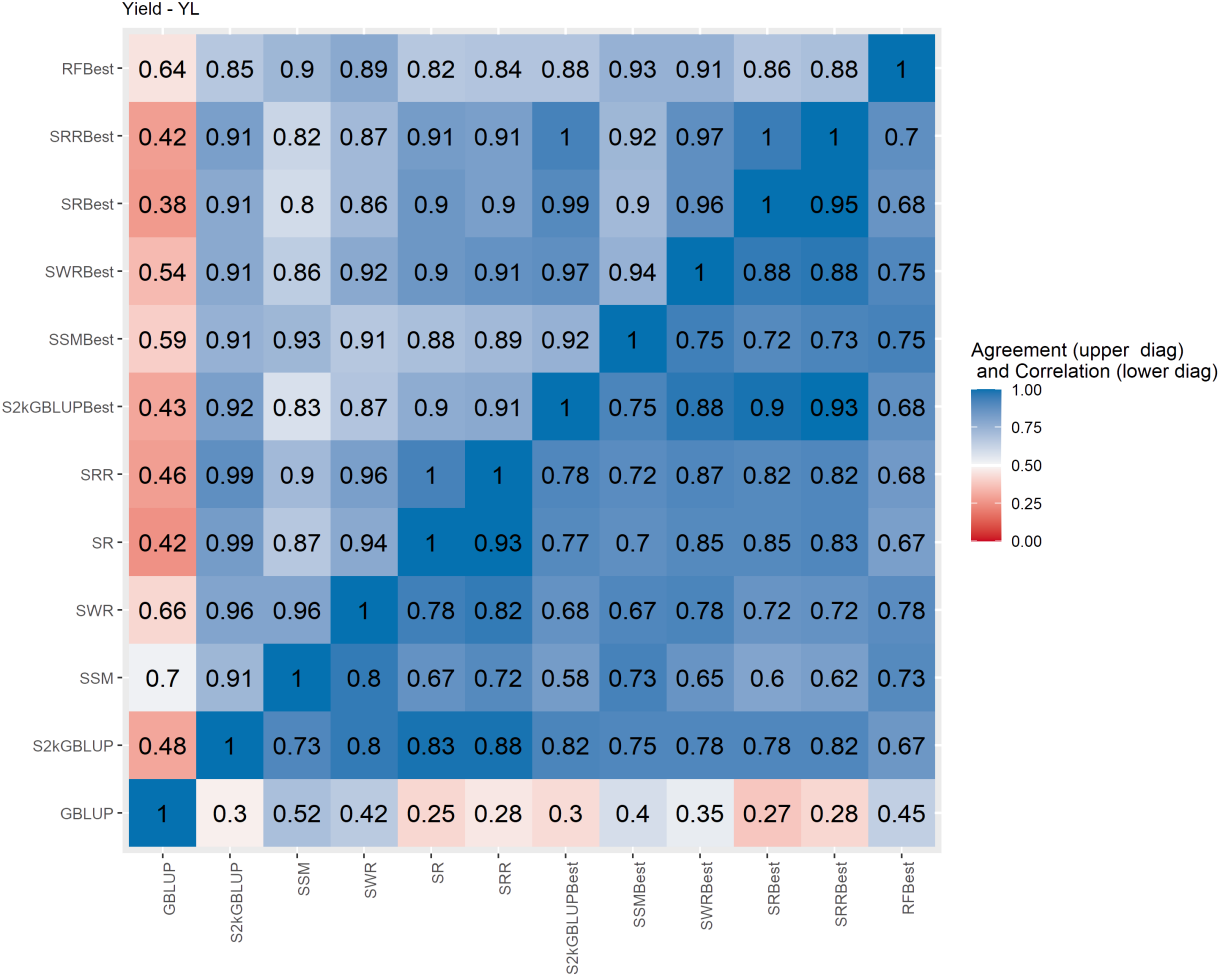
Spearman’s correlation between the genomic estimated breeding values [GEBV] (lower diagonal matrix) and the concordance coefficient between the top 10% of the selected individuals (upper triangular matrix) considering all the different fitted models including the GBLUP model and the meta-learners for yield (YL). The fitted models used as base learners are: Stacking Simple Mean (SSM), Stacking Weighed Regression (SWR), Stacking Regression (SR), Stacking Ridge Regression (SRR), and the Stacking two-kernel GBLUP model (S2KGBLUP). The models named as best (SSMBest, SWRBest, SRBest, SRRBest, S2KGBLUP, and RFBest) used in the fitting only the results provided by those methods that presented predictive ability higher the mean in the Level 0.

The estimated PA ranged from 0.05 (0.01) to 0.32 (0.02) (**Figure 4**). For YL, NF, LM, and Cer, the highest PA values were 0.20 (0.01), 0.32 (0.01), 0.19 (0.01), and 0.27 (0.01), respectively. These results were obtained by implementing SMBest method, which corresponds to the simple mean considering GEBV-BL-Best metadata. The “best” fitted model was SMBest, and it outperformed the PA of the GBLUP model by 87.44% (the ratio between the PA obtained from SMBest model fit and the GBLUP), 37.83%, 199.82%, and 14.59% for YL, NF, LM, and Cer, respectively (**Figure 3**).

Regarding the different data sets used as input in the SEL approach, combining the predicted values obtained from base learners (GEBV-BL) with training data used to fitting the models, did not improve PA of these methods (**Figure 3**). Additionally, the results considering only the predicted values provided from those base learners with PA higher than mean of all base learner (GEBV-BL-Best) as input in the Level 1, returned the highest results (**Figure 3**).

For the four traits, the GBLUP model presented the lowest MSE values (**Supplementary Table S2**). As expected, since the Ridge Regression model (SRR) depends on a regularization parameter it presented a significant higher MSE (**Supplementary Table S2**). For this model, the MSE values were equal to 69.28 (9.18), 8390.68 (518.49), 6.65 (0.80), and 3.45 (0.36) for YL, NF, LM, and Cer, respectively.

**Figure 5:**
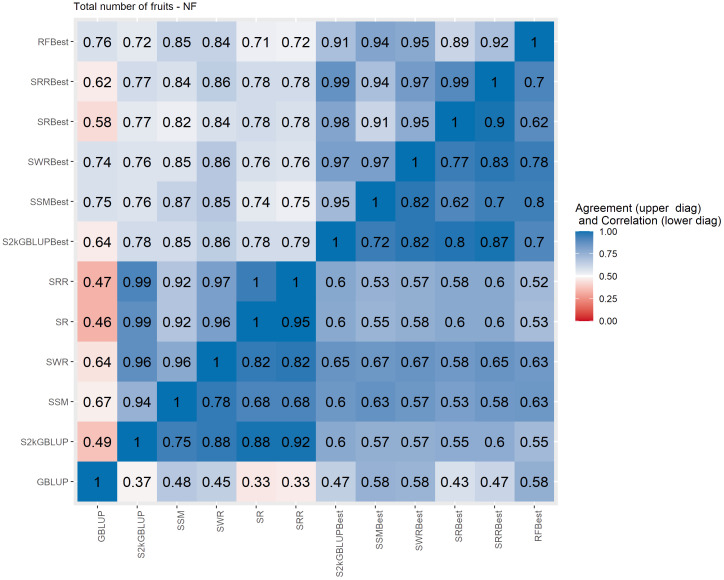
Spearman’s correlation between the genomic estimated breeding values [GEBV] (lower diagonal matrix) and the concordance coefficient between the top 10% of the selected individuals (upper triangular matrix) considering all the different fitted models including the GBLUP model and the meta-learners for total number of fruits (NF). The fitted models used as base learners are Stacking Simple Mean (SSM), Stacking Weighed Regression (SWR), Stacking Regression (SR), Stacking Ridge Regression (SRR), and the Stacking two-kernel GBLUP model (S2KGBLUP). The models named as best (SSMBest, SWRBest, SRBest, SRRBest, S2KGBLUP, and RFBest) used in the fitting only the results provided by those methods that presented predictive ability higher the mean in the Level 0.

**Figure 6:**
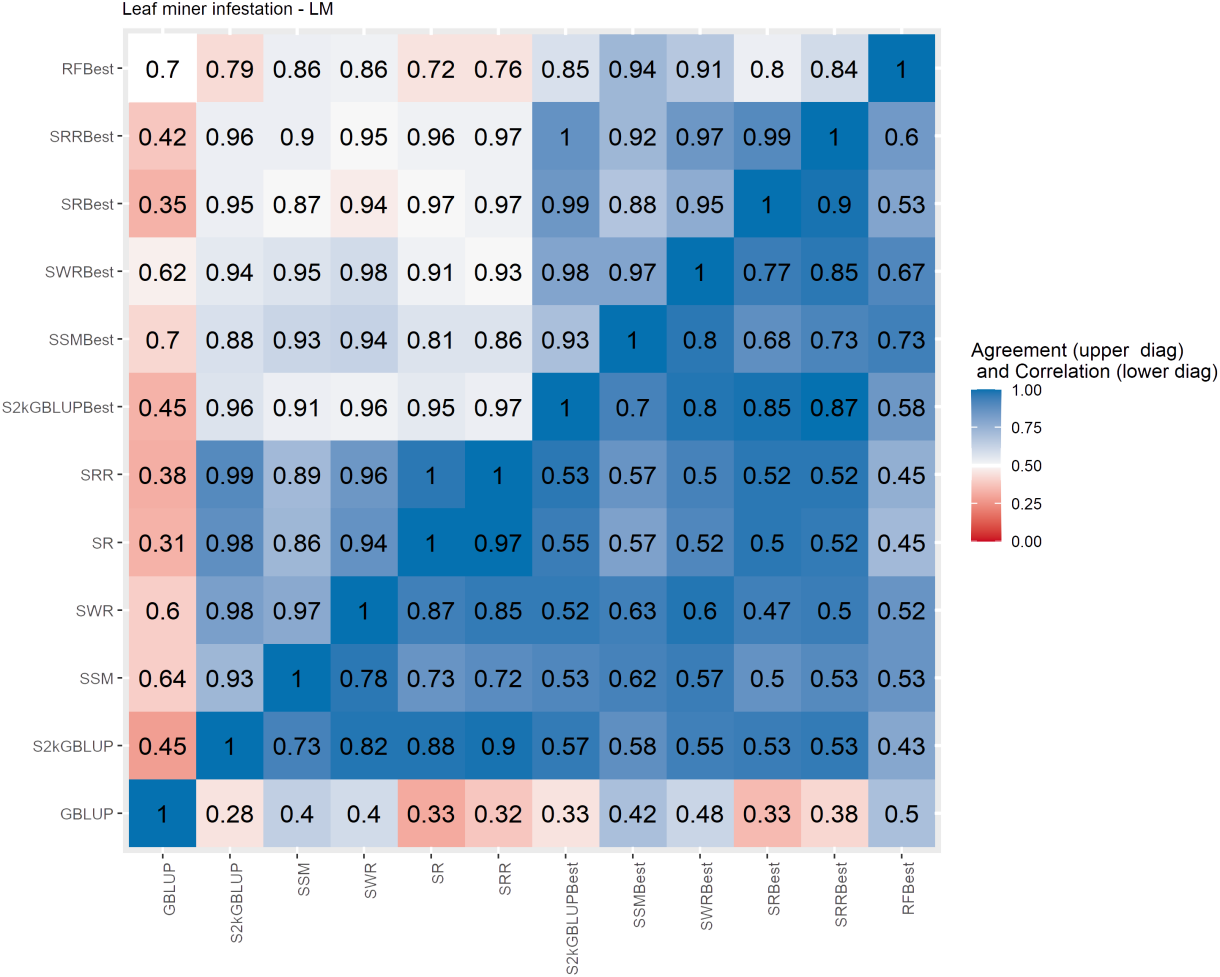
Spearman’s correlation between the genomic estimated breeding values [GEBV] (lower diagonal matrix) and the concordance coefficient between the top 10% of the selected individual’s upper triangular matrix) considering all the different fitted models including the GBLUP model and the meta-learners for leaf minor infestation (LM). The fitted models used as base learners are: Stacking Simple Mean (SSM), Stacking Weighed Regression (SWR), Stacking Regression (SR), Stacking Ridge Regression (SRR), and the Stacking two-kernel GBLUP model (S2KGBLUP). The models named as best (SSMBest, SWRBest, SRBest, SRRBest, S2KGBLUP, and RFBest) used in the fitting only the results provided by those methods that presented predictive ability higher the mean in the Level 0.

**Figure 7:**
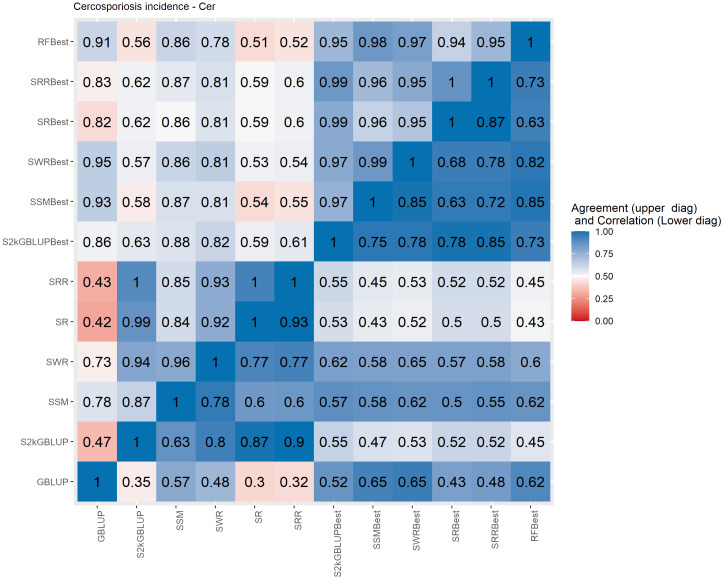
Spearman’s correlation between the genomic estimated breeding values [GEBV] (lower diagonal matrix) and the concordance coefficient between the top 10% of the selected individuals (upper triangular matrix) considering all the different fitted models including the GBLUP model and the meta-learners for Cercosporiosis incidence (Cer). The fitted models used as base learners are: Stacking Simple Mean (SSM), Stacking Weighed Regression (SWR), Stacking Regression (SR), Stacking Ridge Regression (SRR), and the Stacking two-kernel GBLUP model (S2KGBLUP). The models named as best (SSMBest, SWRBest, SRBest, SRRBest, S2KGBLUP, and RFBest) used in the fitting only the results provided by those methods that presented predictive ability higher the mean in the Level 0.

The Spearman’s correlation between the GEBVs obtained with the different prediction models, including the baseline GBLUP model and all the SEL models, presented positive values and these vary from low 0.25 to high 0.97 across the evaluated traits (**Figures 4**–**7**, lower triangular matrix). Low values of the Spearman’s correlation were observed between the GBLUP and the other SEL methods and these were 0.25, 0.33, 0.28, and 0.30 for YL, NF, LM, and Cer, respectively (**Figures 4**–**7**, lower triangle). On the other hand, the highest correlation value (0.97) was observed between the GEBVs obtained by the Stacking Regression (SR) and the Stacking Ridge Regression (SRR) for LM (**Figure 6**).

For each prediction method, the predicted values were ordered based on rankings then the percentage of common individuals in the top 10% between pairs of methods was computed. Overall, the GBLUP presented lower agreement with the SEL evaluated approaches (**Figures 4**–**7**, upper triangle). For instance, the agreement coefficient between the GBLUP and the SRB, SR, SR, and S2KGBLUP methods presented values varying from 0.31 to 0.48 for all evaluated traits (**Figures 4**–**7**, upper triangle).

Regarding the different data sets used as input in the SEL approach, the highest Spearman’s correlations and agreements were observed between those methods that used the same kind of metadata as input in the fitting. Overall, considering these two measures, the methods were grouped into three groups (**Supplementary Figures S6**-**S13**). In general, the GBLUP was allocated into a single group. The only exception was for cercorporiosis (Cer) considering the agreement measure. Is this case, the GBLUP was allocated together with those methods that’s considers only the predicted values provided from those base learners with PA higher than the mean of all base learner (GEBV-BL-Best) as input in the SEL approaches (**Supplementary Figure S12**).

## 4 Discussion

In this study, we used the SEL approach to improve PA of four important traits in Coffea Arabica. Two of these traits are associated with the productivity (YL and NF) and the remaining two with disease resistance (LM and Cer). The population under study is comprise of 195 genotypes of Coffea Arabica genotyped for 5,970 SNP markers. We compared the PA of different approaches used in the Level 1 of the SEL to the results obtained with the base learners [GBLUP, MARS considering degrees equal to 1, 2, and 3, QRF considering nine quantiles (from 0.1 to 0.9, every 0.1) and RF]. Since the GBLUP is the most implemented prediction model (Zhang et al., 2021), their results were used as benchmark. The PA of the different approaches was assessed using a CV scheme repeated 10 times. The Spearman’s correlation and the agreement (based on the top 10%) coefficients between the GEBV values of the different models were also estimated. The genetic parameters were also estimated for the evaluated traits (YL, NF, LM, and Cer).

The heritability estimates for YL (0.55), NF (0.49), LM (0.30), and Cer (0.38) were consistent with those reported in the literature for this specie and same traits. Specifically, the heritability estimates varied between (0.1–0.74) [Alkimim et al. (2020) and Alemayehu, 2019], (0.30–0.55) [Gokavi et al., 2023 and Weldemichael et al., 2017], (0.30–0.51) [Chrigui et al., 2020 and Ferrão et al., 2023], and (0.09–0.61) Alkimim et al., 2021 and Ferrão et al., 2023] for the YL, NF, LM, and Cer, respectively. Although the Pearson correlation between YL and the disease resistance traits were not statistically significant, a significant and positive genetic correlation (0.52) was obtained between YL and NF.

The machine learning methods as base learners have been already used in genomic prediction (Long et al., 2011; Lenz et al., 2019; Montesinos-López et al., 2019; Coelho de Sousa et al., 2022; Costa et al., 2022). However, generally these methods do not outperform significantly the traditional genomic prediction approach based on parametric models such as GBLUP and Bayesian Alphabet (Liang et al., 2021).

Liang et al. (2021) used the SEL for improving PA in three real datasets on average by 7.70%, compared to GBLUP. The SEL uses predicted values from different machine learning implementations to obtain a single prediction value. These authors integrated/combined the results of three machine learning implementations (Support Vector Machine, Kernel Ridge Regression and Elastic Net) to compute the GEBVs.

In contrast to Liang et al. (2021), in our study, the GBLUP approach was used as one of the base learner methods for the SEL too. The GBLUP was considered since it is widely used for genome prediction (Zhang et al., 2021) due to its reduced computational demand and simplicity (Hernandez et al., 2020) compared to the other parametric methods (e.g., Bayesian Alphabet, Gianola et al., 2009). The MARS that allows automatically selecting and modeling nonlinear relationships and interaction effects of the predictor variables was also considered as base-learner method (Costa et al., 2022). In addition, the RF (James et al., 2023) and the QRF (Meinshausen, 2006) were also set as base learner methods. Specifically, the RF is a machine learning approach used to increase the predictive power and reduce the variance of the predicted values by averaging uncorrelated quantities (James et al., 2023). The QRF combines interesting characteristics from RF and Quantile Regression (QR) approaches. QR (Koenker and Bassett, 1978) allows fitting all the portions of the probability distribution of the trait, enabling a more complete picture of the conditional distribution than a single estimate of the center (Briollais and Durrieu, 2014; Nascimento et al., 2019).

Overall, for each evaluated trait (YL, NF, LM, and Cer), a different model presented the highest PA. These results show that there is not a single approach that outperforms the others in the evaluated data sets. Also, it could be case of a model performing better than the others in a given dataset but another model could perform better in a similar dataset (James et al., 2023). For example, the MARS models with 2° and 3° (model with interactions) presented higher PA for YL. These results are in line with those obtained by Coelho de Sousa et al. (2022). Using artificial neural networks to predict the genetic merit of genotypes of *Coffea canephora* these authors showed a larger dominance markers effect for YL when compared to the GBLUP additive dominant model based on additive marker effects.

Another interesting result was obtained modeling the QRF where the distribution of the adjusted phenotypic values for YL and NF. These presented a higher concentration in the first quantiles (0.1–0.3) (**Supplementary Figure S2**) and positive skewness (**Supplementary Figure S3**). For YL, the best models were the QRF0.1 and QRF0.2, and QRF0.3 for NF (**Supplementary Figure S2**). The distribution of the adjusted LM values presented tree major portions (**Supplementary Figure S4**). The QRF modeling was able to distinguish these three different groups (**Figure 2**). Finally, since the distribution of adjusted Cer phenotypic values did no present a specific pattern to highlight (**Supplementary Figure S5**) all of the QRF models present similar PA (**Figure 2**). A similar trend was shown by Nascimento et al. (2017). These authors found that the Quantile Regression approach outperform the traditional genomic prediction methods of not normal distributed traits.

An interesting approach to address the non-normality assumption is using multiple models to conduct the predictions, and then combine the predicted values to makeup a single prediction through the SEL approach. In general, the SEL outperforms the methods based on base learners only (Liang et al., 2021; Kandel et al, 2021; Kalule et al., 2023). In our study, the SEL approach outperformed all base learner methodologies (**Figures 2**, **3**). However, it is important to emphasize that these results were observed by those SEL models that used only the predicted values provided from the base learners with PA higher than mean of all the base learners. Specifically, the Stacking Mean Best (SMB) presented the highest PA for all of the evaluated traits. The average of the predictions from several fitted models has been successfully implemented with Bagging and RF approaches (Breiman, 1996 and Breiman, 2001). The SEL approach allow to use several models to combine the predicted values, for example, XGBoost (Ghasemieh et al., 2023), Penalized methods (Kalule et al., 2023), Linear Regression (Liang et al., 2021). Similar to the single model approach, the performance of the different SEL implementation can vary from one data set to another with no one of these outperforming the others in all data sets. Thus, as it was recommended by these authors it is important to evaluate several models as meta-learners as well.

Regarding the MSE, as expected, the penalized models, showed larger values compared to the other evaluated methods. By design these methods induce bias aiming to reduce the variance of the estimations (Montgomery et al., 2021; Chan et al., 2022). However, these cannot guarantee the increasing of the PA compared to other methods. The SMB, which resulted to return the best results in terms of PA, also presented large values for the MSE. This can be a consequence that SMB-SEL uses predicted values derived from base learners that return large MSE values (**Supplementary Table S1**).

Overall, the SEL models presented moderate to high Spearman’s correlation between them (**Figures 4**–**6**). On the other hand, these were low to moderate between SEL approaches and the GBLUP model. Additionally, among the 10% of genotypes with the highest GEBVs for YL, NF, LM, and Cer, the agreement coefficient between the SEL and GBLUP models showed values varying from moderate to high, suggesting differences in the obtained classifications with these. In general, the cluster analysis of these results showed that the methods can be grouped into three distinct groups (**Supplementary Figures S6**-**S13**) with the GBLUP forming a group by itself.

Altogether, these results show that the use of SEL to predict the individual genetic merit of four important traits in Arabica Coffee is worth to investigate. The SEL approach showed higher estimates of PA compared with all evaluated base learning methods, in special to the traditional GBLUP method. In practice, SEL’s ability to combine methods with diverse characteristics facilitates a more comprehensive exploration of the relationships between variables leading to more accurate selection of breeding. This approach considers a wider range of factors and reduces the reliance on any single model’s limitations. However, evaluating phenotypes across multiple environments can pose challenges for SEL. Unlike GBLUP, which presents higher computational efficiency (Hernandez et al., 2020), many base learners in SEL methods are based on machine learning requiring significant computation time in certain scenarios. Studies have explored the use of single machine learning methods for multi-environment trials (METs). For example, Barreto et al. (2024) applied machine learning to predict hybrid performance in METs and achieved similar PA compared to GBLUP with non-additive effects. As highlighted by Montesinos López et al. (2022) in their study using RF for METs, training any machine learning model can be computationally demanding, especially for datasets where the training data sets are very large. The hyperparameter tuning for individual base learners within a SEL framework is a well-established approach to enhance model performance. However, it is important to acknowledge that SEL ensembles can achieve strong results even with default base learner parameters (Friedel et al., 2023). This aligns perfectly with the core principle of ensemble learning, that is, leveraging predictions from multiple models can outperform any single model. In our study, the high dimensionality of the data presented significant computational challenges for hyperparameter tuning. Additionally, the observed superiority of the SEL approach compared to traditional methods suggested that tuning might not be as critical for achieving good results.

## 5 Conclusion

The SEL method was able to predict the PA of important traits (YL, NF, leaf miner infesting, cercosporiosis resistance) in Coffea Arabica. In addition, SEL presented higher PA compared with those obtained for all base learner methods (GBLUP, MARS considering degrees equal to 1, 2, and 3, QRF considering nine quantiles, from 0.1 to 0.9, every 0.1 and RF).

## Data availability statement

The original contributions presented in the study are included in the article/**Supplementary Material**. Further inquiries can be directed to the corresponding authors.

## Author contributions

MN: Writing – review & editing, Writing – original draft, Validation, Software, Methodology, Investigation, Formal analysis, Conceptualization. AN: Writing – review & editing, Software, Methodology, Investigation. CA: Writing – review & editing, Methodology. AO: Writing – review & editing, Data curation. EC: Writing – review & editing, Investigation, Data curation. DJ: Writing – review & editing, Methodology, Investigation.
