# Supplementary material for: Enhancing genomic prediction with Stacking Ensemble Learning in Arabica Coffee
Source: Front Plant Sci. 2024 Jul 17;15:1373318. doi: 10.3389/fpls.2024.1373318 (PMC11288849; doi:10.3389/fpls.2024.1373318)
Supplement: Supplementary file 1 [file DataSheet_1.docx]

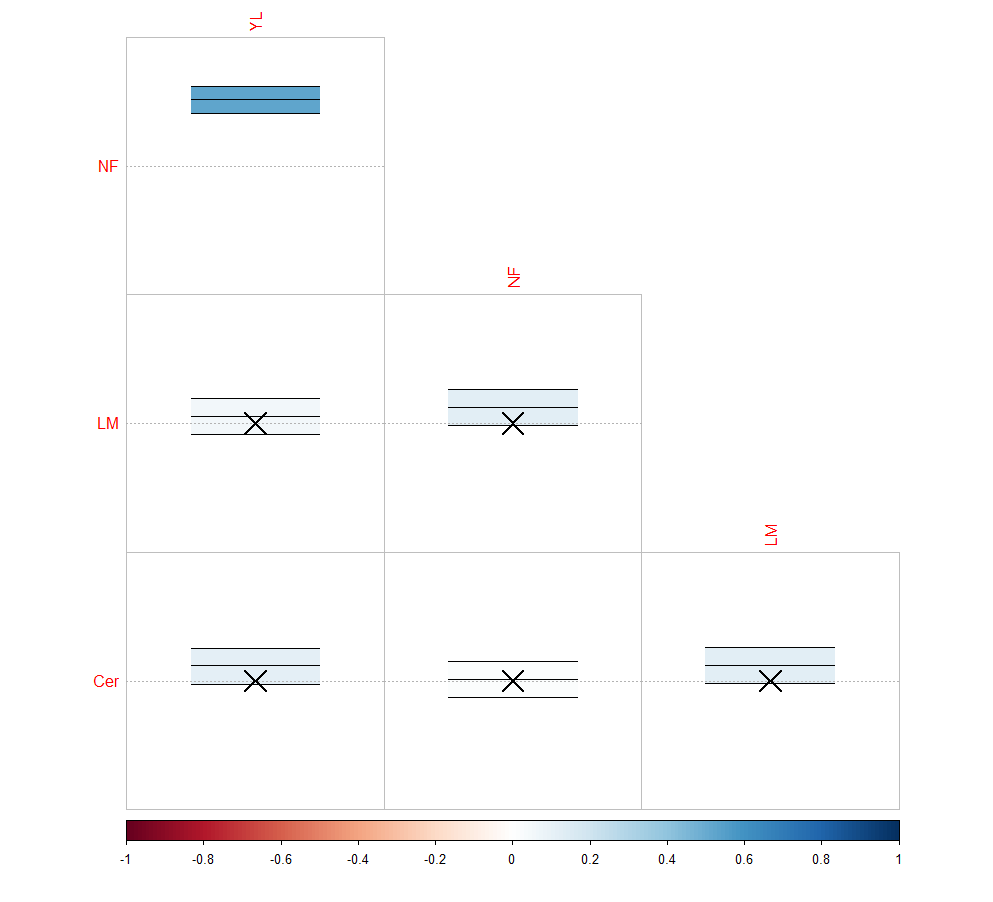


**Figure S1.** Lower and upper bound of the 95% confidence interval of the Spearman’s correlation parameter between the adjusted phenotypic values of the different traits: YL, yield; NF, total number of fruits; LM, leaf miner infestation; and Cer, Cercosporiosis incidence. The horizontal dashed line is centered on zero. The “X” signal in the figure indicates there is not statistical evidence that the correlation is different from zero.


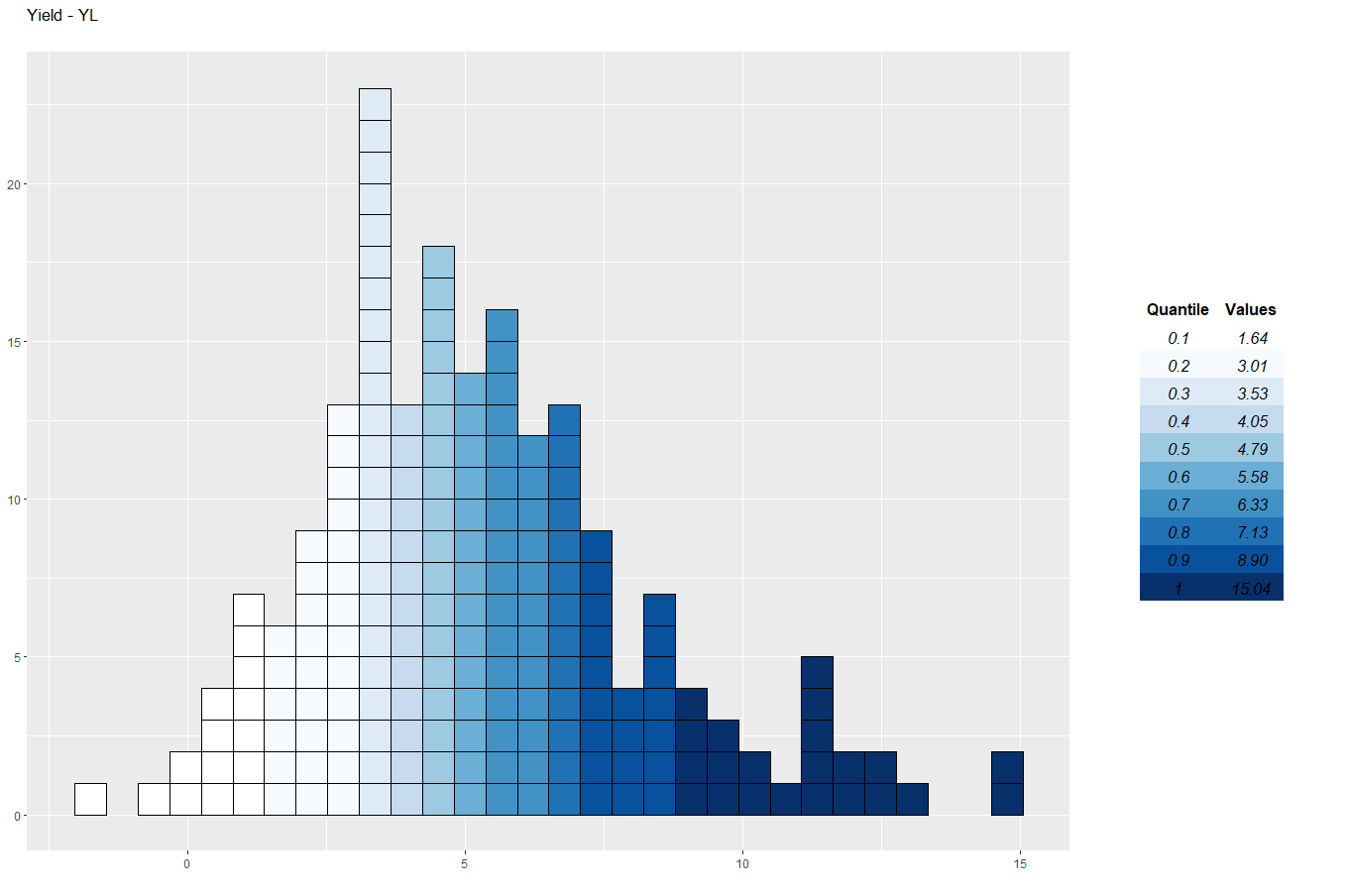


**Figure S2.** Histogram and quantiles of adjusted phenotypic values for yield (YL).


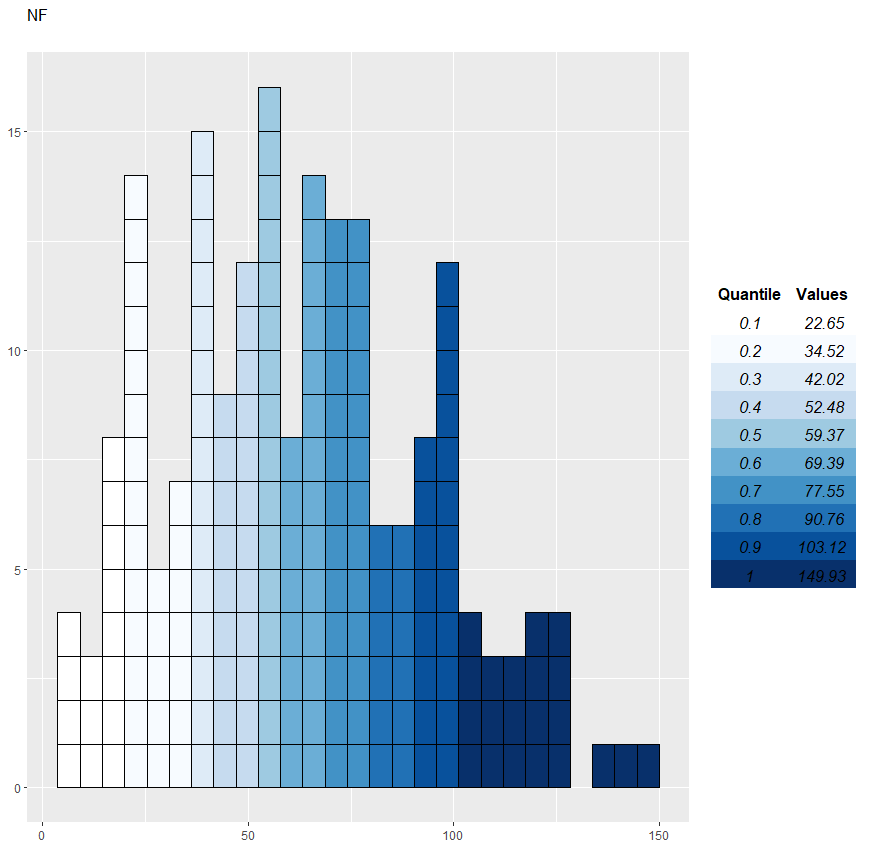


**Figure S3.** Histogram and quantiles of adjusted phenotypic values for Number of fruits (NF).


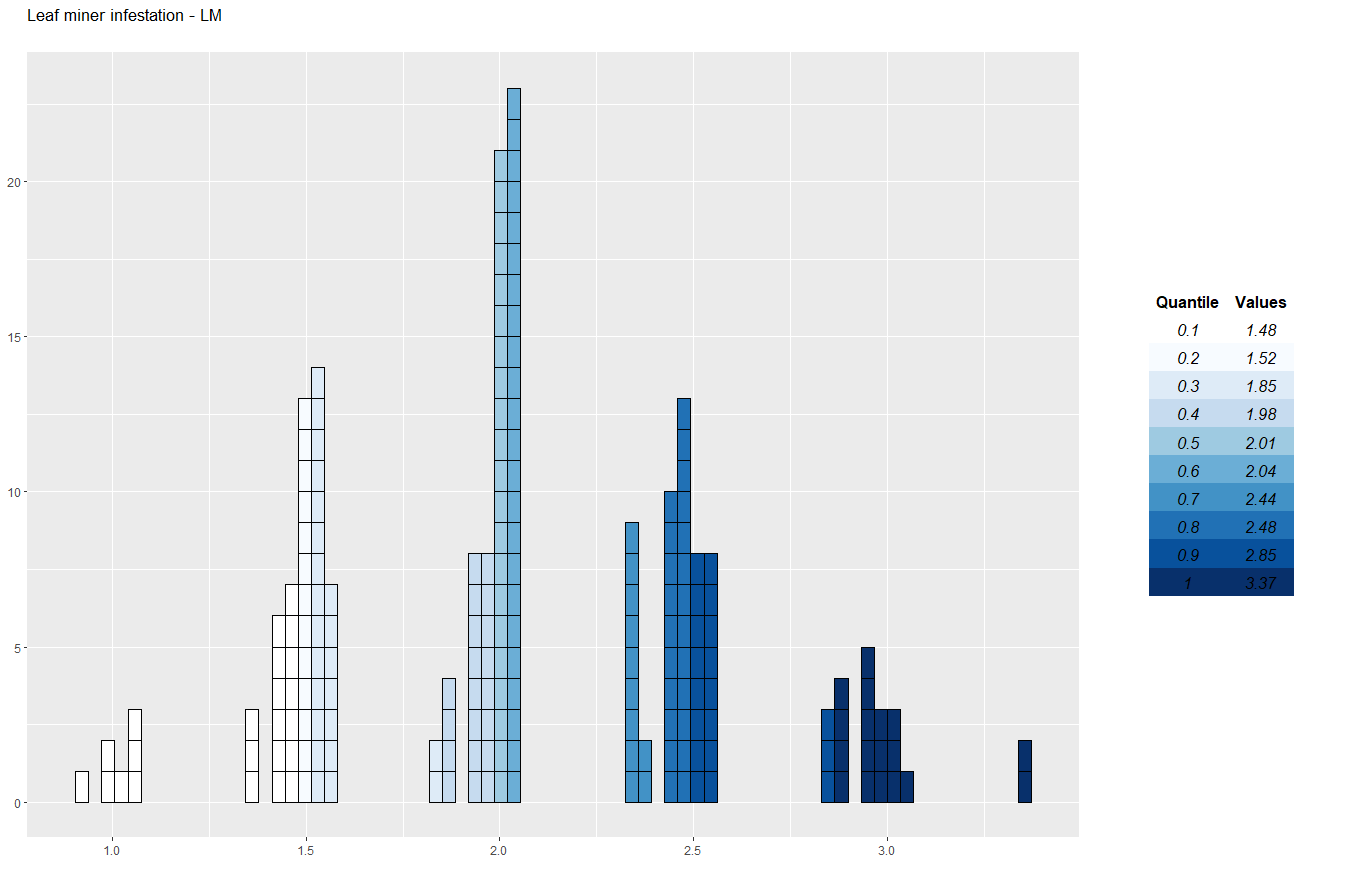


**Figure S4.** Histogram and quantiles of adjusted phenotypic values for Leaf miner infestation (LM).


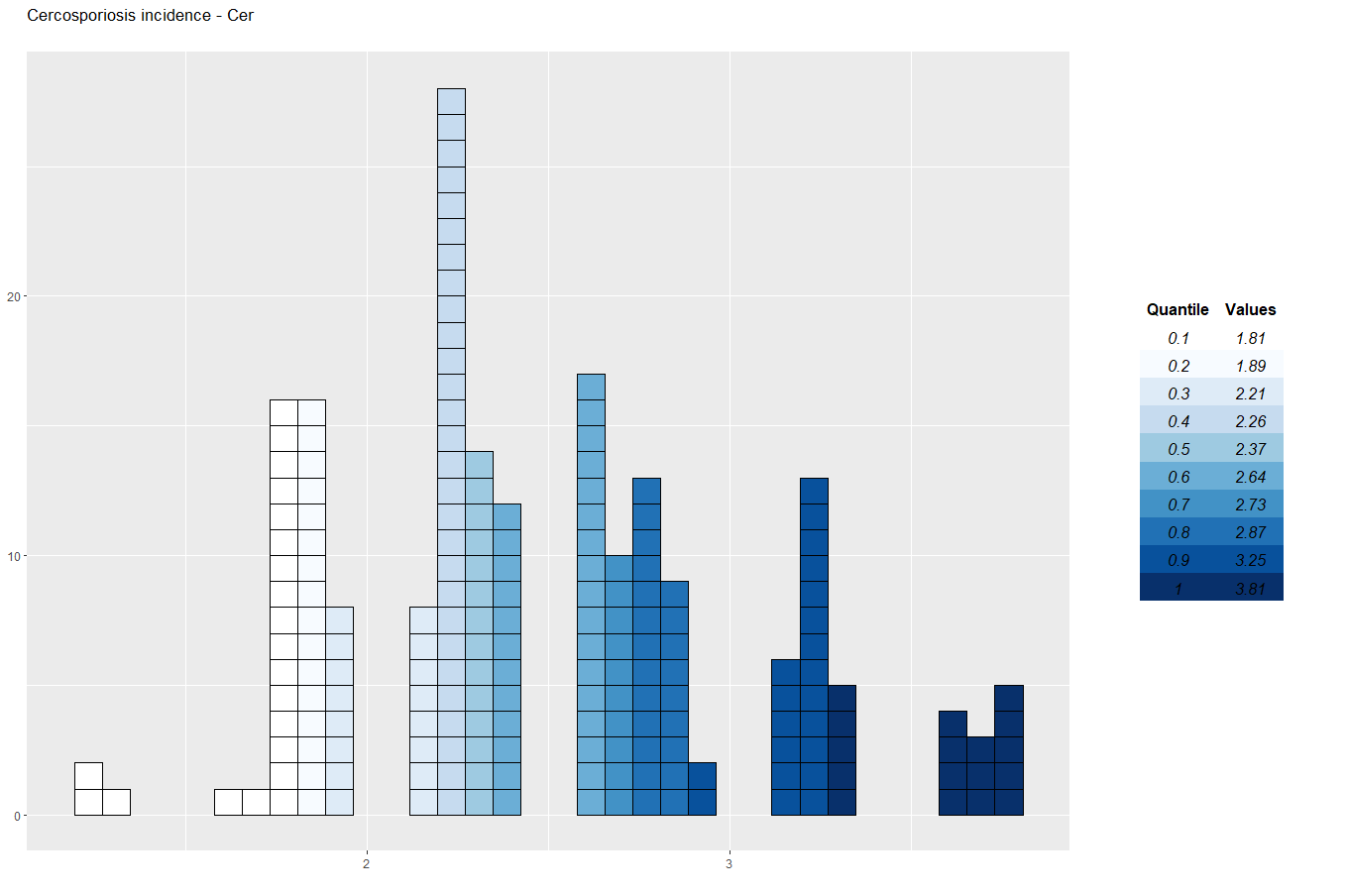


**Figure S5.** Histogram and quantiles of adjusted phenotypic values for Cercosporiosis incidence (Cer).


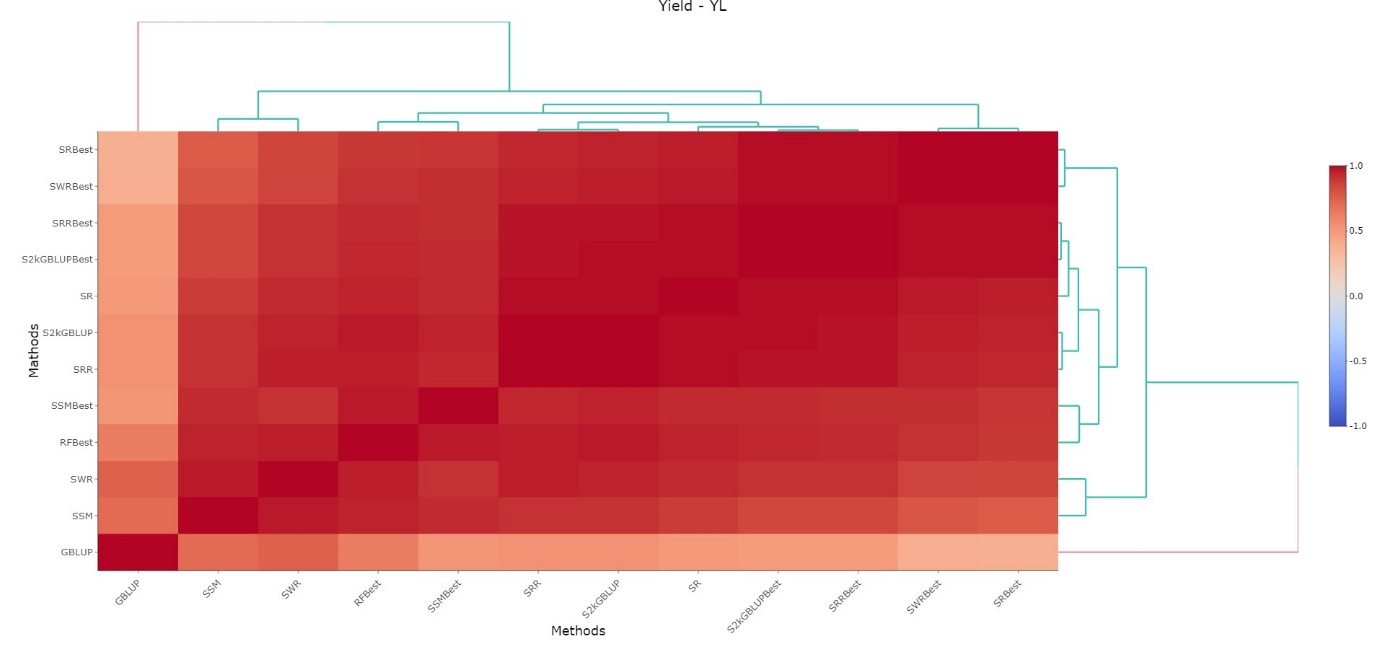


**Figure S6.** Heatmap and cluster analysis of the estimates of Spearman’s correlation between the genomic estimated breeding value obtained considering Genomic BLUP and all the fitted models used as meta learners for Yield measured 195 individuals of arabica coffee. The fitted models used as base learners are: Stacking Simple Mean (SSM), Stacking Weighed Regression (SWR), Stacking Regression (SR), Stacking Ridge Regression (SRR) and, the Stacking two kernel GBLUP model (S2KGBLUP). The models named as best (SSMBest, SWRBest, SRBest, SRRBest, S2KGBLUP and, RFBest) used in the fitting only the results provided by those methods that presented predictive ability higher the mean in the level 0.


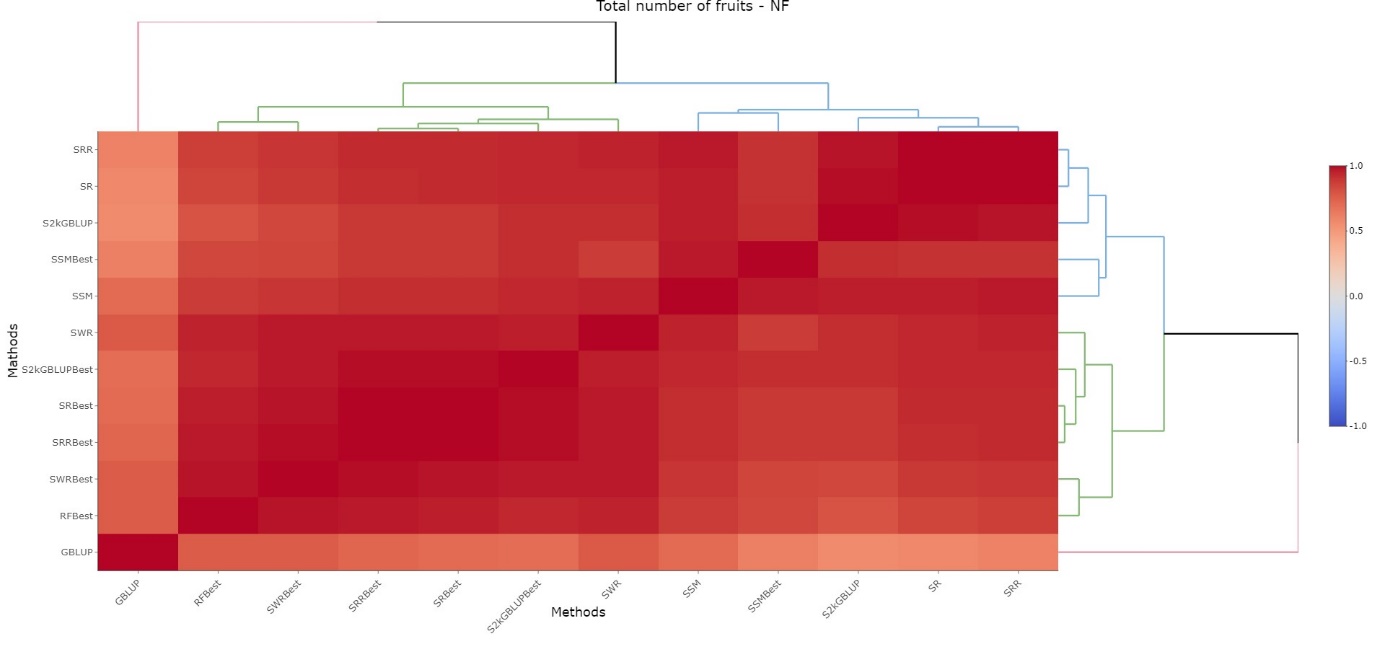


**Figure S7.** Heatmap and cluster analysis of the estimates of Spearman’s correlation between the genomic estimated breeding value obtained considering Genomic BLUP and all the fitted models used as meta learners for Total number of fruits measured 195 individuals of arabica coffee. The fitted models used as base learners are: Stacking Simple Mean (SSM), Stacking Weighed Regression (SWR), Stacking Regression (SR), Stacking Ridge Regression (SRR) and, the Stacking two kernel GBLUP model (S2KGBLUP). The models named as best (SSMBest, SWRBest, SRBest, SRRBest, S2KGBLUP and, RFBest) used in the fitting only the results provided by those methods that presented predictive ability higher the mean in the level 0.


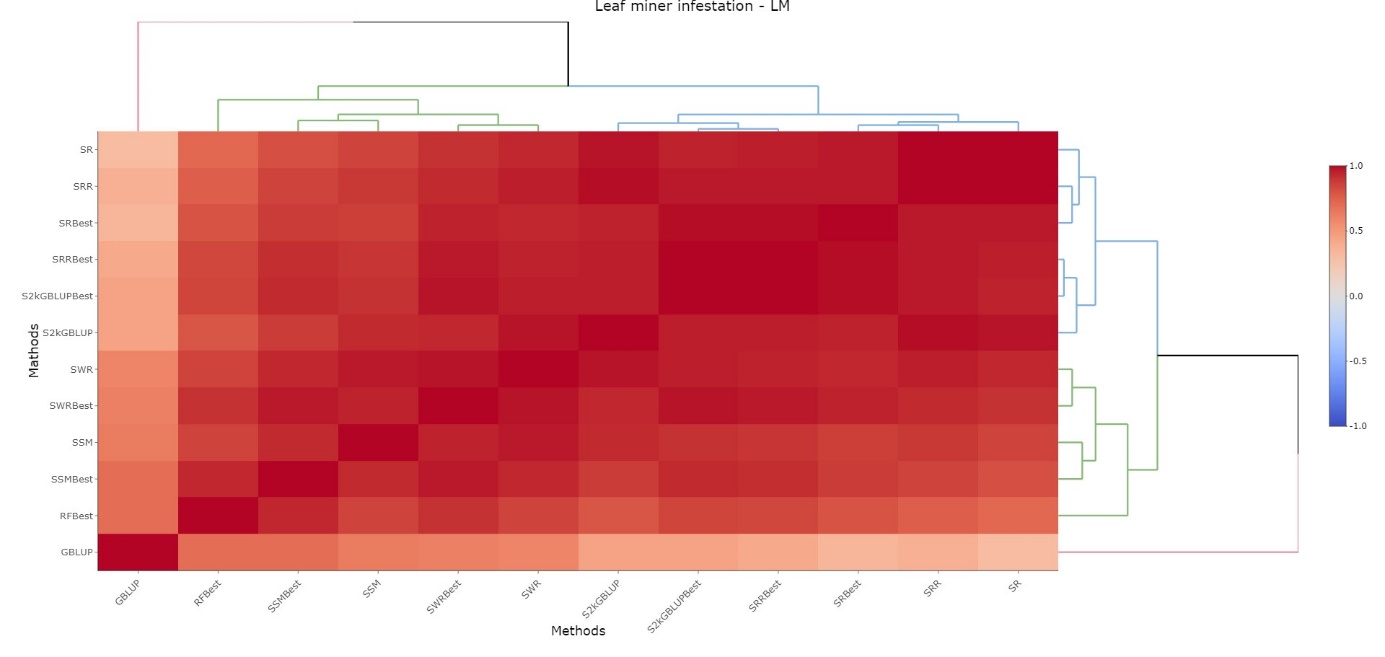


**Figure S8.** Heatmap and cluster analysis of the estimates of Spearman’s correlation between the genomic estimated breeding value obtained considering Genomic BLUP and all the fitted models used as meta learners for Leaf miner infestation measured 195 individuals of arabica coffee. The fitted models used as base learners are: Stacking Simple Mean (SSM), Stacking Weighed Regression (SWR), Stacking Regression (SR), Stacking Ridge Regression (SRR) and, the Stacking two kernel GBLUP model (S2KGBLUP). The models named as best (SSMBest, SWRBest, SRBest, SRRBest, S2KGBLUP and, RFBest) used in the fitting only the results provided by those methods that presented predictive ability higher the mean in the level 0.


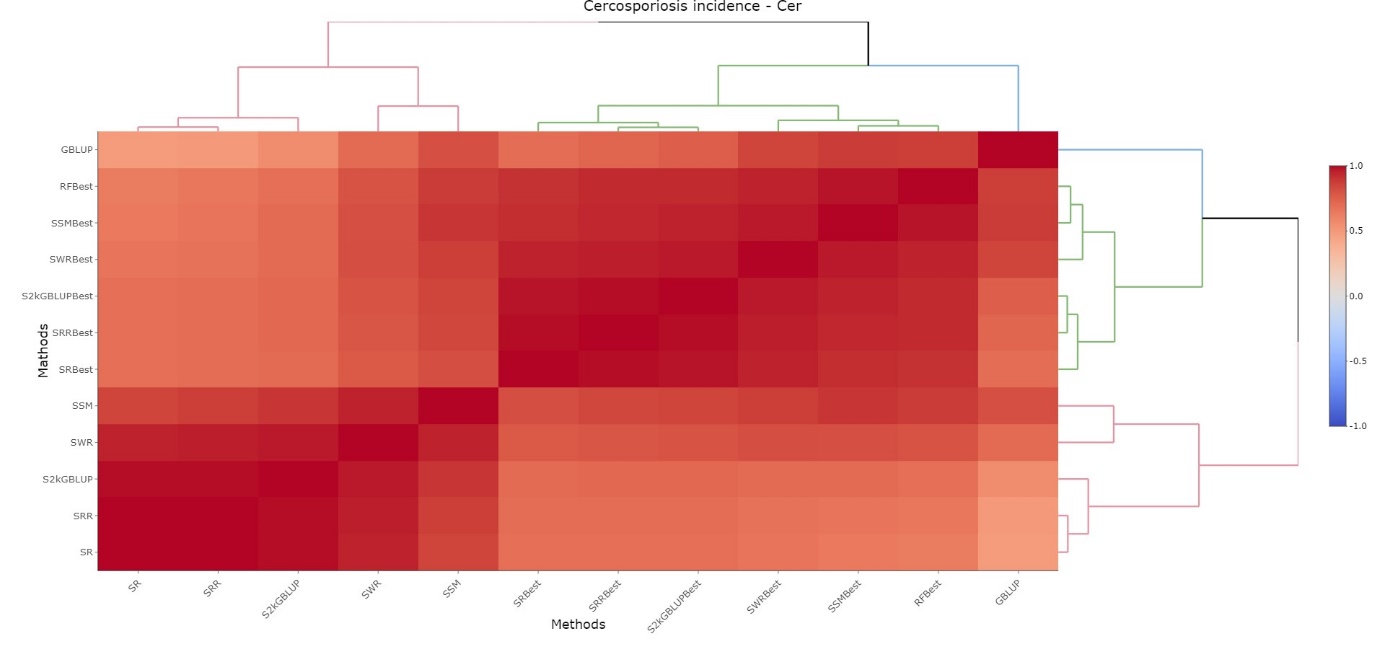


**Figure S9.** Heatmap and cluster analysis of the estimates of Spearman’s correlation between the genomic estimated breeding value obtained considering Genomic BLUP and all the fitted models used as meta learners for Cercosporiosis incidence measured 195 individuals of arabica coffee. The fitted models used as base learners are: Stacking Simple Mean (SSM), Stacking Weighed Regression (SWR), Stacking Regression (SR), Stacking Ridge Regression (SRR) and, the Stacking two kernel GBLUP model (S2KGBLUP). The models named as best (SSMBest, SWRBest, SRBest, SRRBest, S2KGBLUP and, RFBest) used in the fitting only the results provided by those methods that presented predictive ability higher the mean in the level 0.


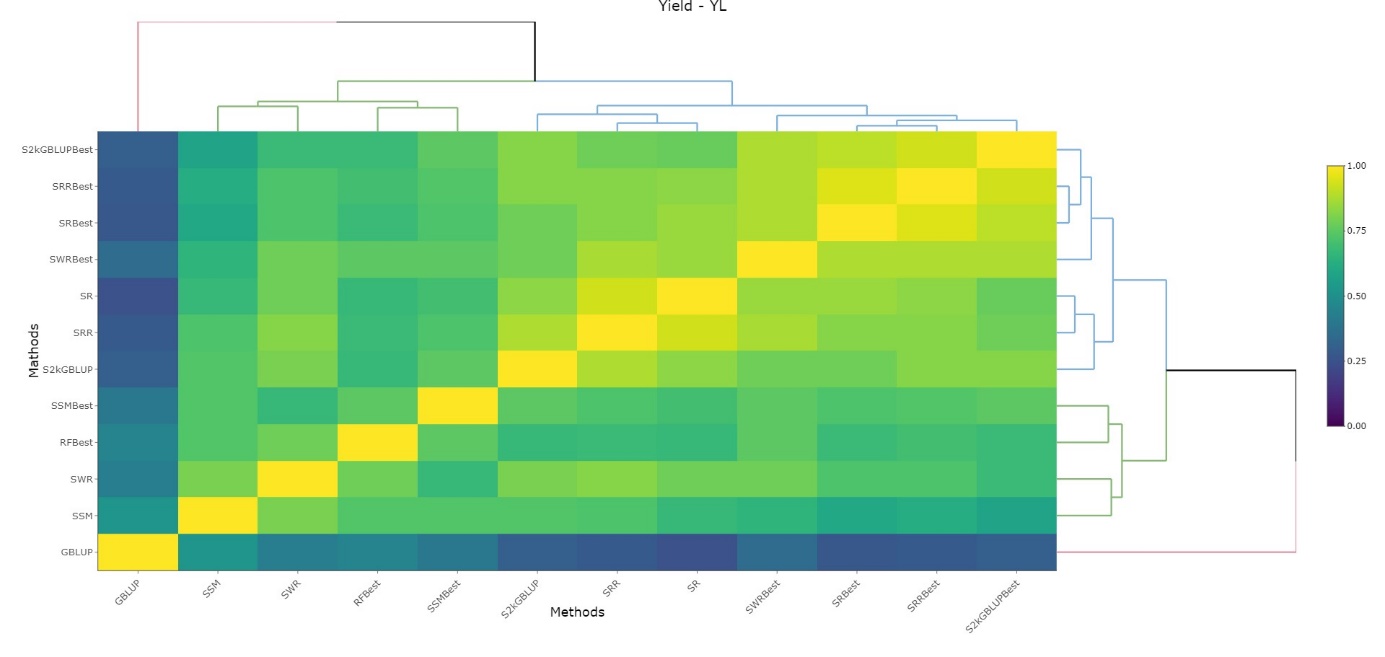


**Figure S10.** Heatmap and cluster analysis of the concordance coefficient between the 10% selected individuals considering the GEBV obtained considering Genomic BLUP and all the fitted models used as meta learners for Yield measured 195 individuals of arabica coffee. The fitted models used as base learners are: Stacking Simple Mean (SSM), Stacking Weighed Regression (SWR), Stacking Regression (SR), Stacking Ridge Regression (SRR) and, the Stacking two kernel GBLUP model (S2KGBLUP). The models named as best (SSMBest, SWRBest, SRBest, SRRBest, S2KGBLUP and, RFBest) used in the fitting only the results provided by those methods that presented predictive ability higher the mean in the level 0.


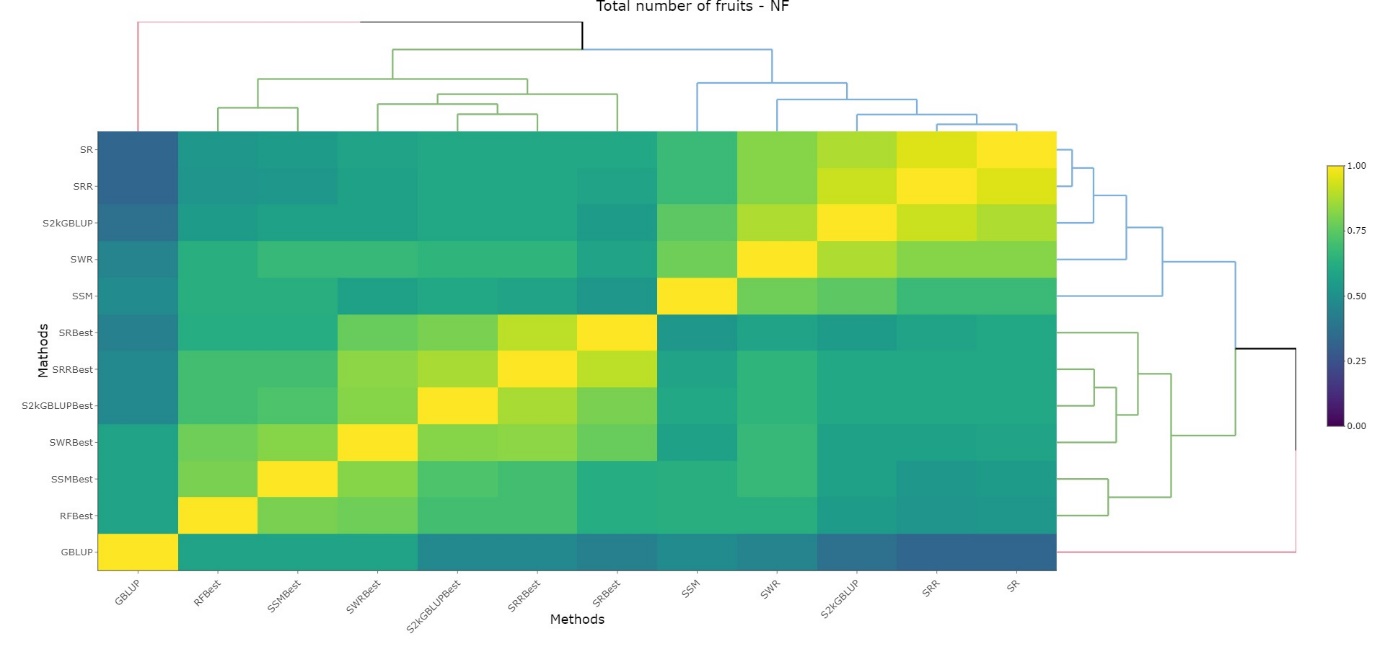


**Figure S11.** Heatmap and cluster analysis of the concordance coefficient between the 10% selected individuals considering the GEBV obtained considering Genomic BLUP and all the fitted models used as meta learners for Total number of fruits measured 195 individuals of arabica coffee. The fitted models used as base learners are: Stacking Simple Mean (SSM), Stacking Weighed Regression (SWR), Stacking Regression (SR), Stacking Ridge Regression (SRR) and, the Stacking two kernel GBLUP model (S2KGBLUP). The models named as best (SSMBest, SWRBest, SRBest, SRRBest, S2KGBLUP and, RFBest) used in the fitting only the results provided by those methods that presented predictive ability higher the mean in the level 0.


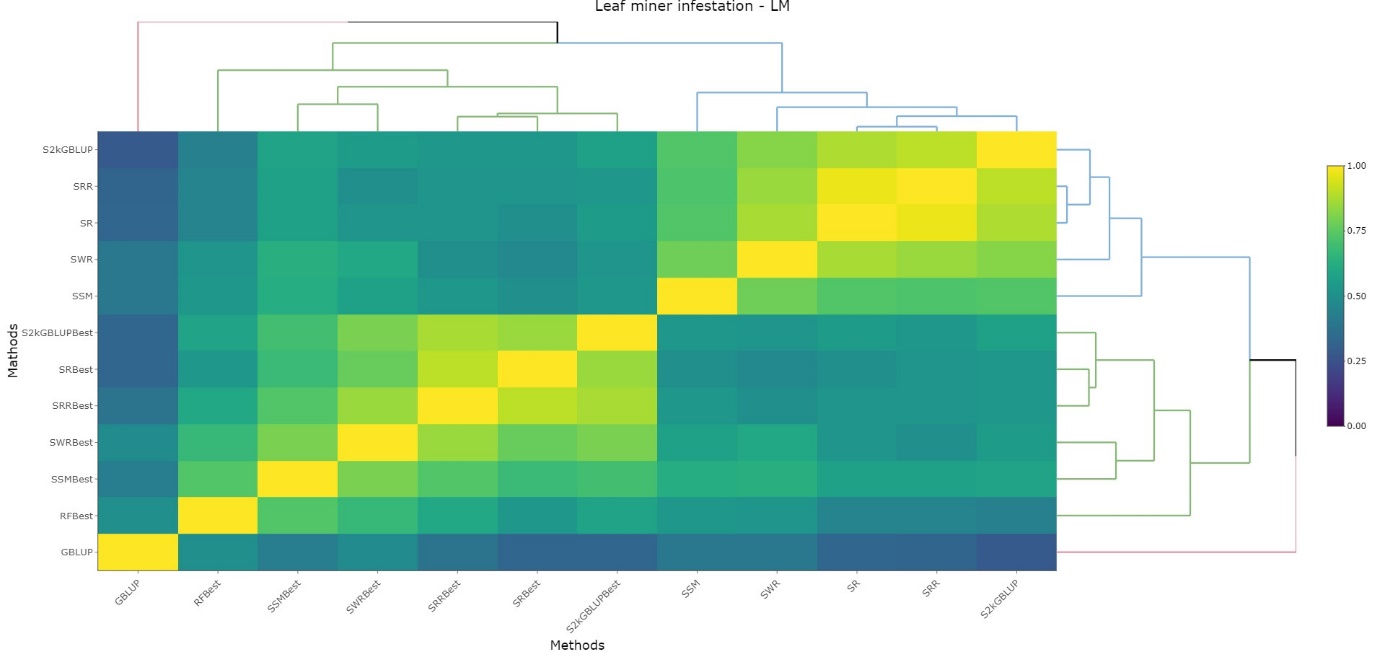


**Figure S12.** Heatmap and cluster analysis of the concordance coefficient between the 10% selected individuals considering the GEBV obtained considering Genomic BLUP and all the fitted models used as meta learners for Leaf miner infestation measured 195 individuals of arabica coffee. The fitted models used as base learners are: Stacking Simple Mean (SSM), Stacking Weighed Regression (SWR), Stacking Regression (SR), Stacking Ridge Regression (SRR) and, the Stacking two kernel GBLUP model (S2KGBLUP). The models named as best (SSMBest, SWRBest, SRBest, SRRBest, S2KGBLUP and, RFBest) used in the fitting only the results provided by those methods that presented predictive ability higher the mean in the level 0.


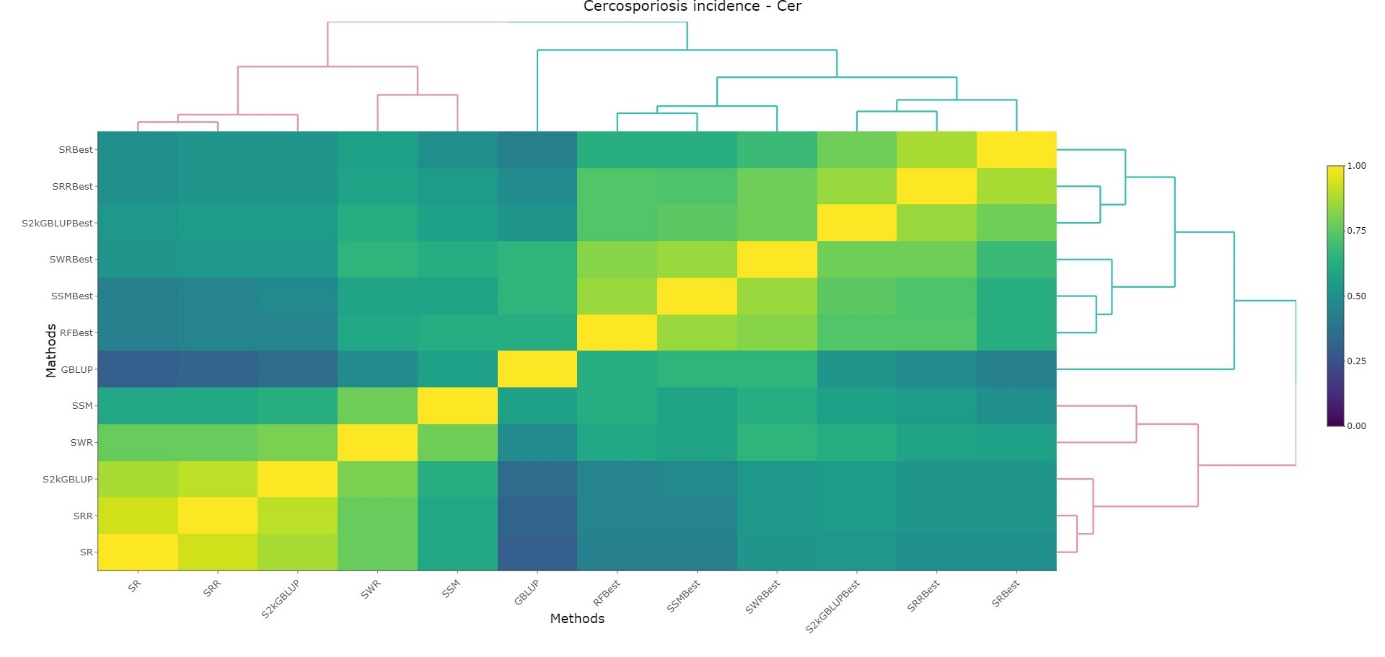


**Figure S13.** Heatmap and cluster analysis of the concordance coefficient between the 10% selected individuals considering the GEBV obtained considering Genomic BLUP and all the fitted models used as meta learners for Cercosporiosis incidence measured 195 individuals of arabica coffee. The fitted models used as base learners are: Stacking Simple Mean (SSM), Stacking Weighed Regression (SWR), Stacking Regression (SR), Stacking Ridge Regression (SRR) and, the Stacking two kernel GBLUP model (S2KGBLUP). The models named as best (SSMBest, SWRBest, SRBest, SRRBest, S2KGBLUP and, RFBest) used in the fitting only the results provided by those methods that presented predictive ability higher the mean in the level 0.

**Table S1.** Mean square error (MSE) for yield (YL), total number of fruits (NF), leaf miner infestation (LM), and cercosporiosis incidence (Cer) measured in an arabica coffee population comprised of 195 individuals using a holdout validation scheme repeated 10 times.

| Methods | Traits | | | |
| --- | --- | --- | --- | --- |
|  | YL | NF | LM | Cer |
| GBLUP | 16.05 (1.01) | 2329.00 (368.99) | 0.21 (0.02) | 0.57 (0.07) |
| MARS 1 | 30.43 (2.81) | 3406.88 (419.85) | 0.30 (0.03) | 1.60 (0.19) |
| MARS 2 | 26.78 (5.29) | 3514.07 (311.67) | 0.70 (0.08) | 1.16 (0.15) |
| MARS 3 | 30.24 (3.49) | 1837.53 (163.18) | 0.71 (0.1) | 1.43 (0.18) |
| QRF0.1 | 718.30 (22.16) | 86641.17 (2526.57) | 19.70 (0.37) | 22.33 (0.79) |
| QRF0.2 | 335.11 (15.25) | 45840.51 (2017.23) | 13.52 (0.34) | 11.23 (0.45) |
| QRF0.3 | 176.78 (10.38) | 19777.36 (1405.39) | 4.45 (0.26) | 5.26 (0.32) |
| QRF0.4 | 81.34 (7.00) | 6573.93 (893.84) | 0.93 (0.07) | 1.33 (0.14) |
| QRF0.5 | 26.75 (2.58) | 2363.44 (403.07) | 0.23 (0.02) | 0.36 (0.05) |
| QRF0.6 | 33.24 (3.74) | 5372.44 (431.05) | 0.74 (0.08) | 1.62 (0.15) |
| QRF0.7 | 126.19 (10.07) | 16979.60 (1084.06) | 3.99 (0.18) | 5.84 (0.28) |
| QRF0.8 | 375.17 (18.16) | 41978.48 (2053.53) | 8.73 (0.21) | 17.04 (0.48) |
| QRF0.9 | 1184.18 (31.06) | 92137.07 (3161.05) | 21.79 (0.47) | 42.48 (0.57) |
| RF | 20.00 (1.69) | 2251.51 (286.18) | 0.20 (0.02) | 0.59 (0.08) |

Genomic Best Linear Unbiased Predictor – (GBLUP); Multivariate Adaptive Regression Splines with degrees equal to 1, 2 and 3 – (MARS 1, MARS 2 and MARS 3); Quantile Random Forest evaluated at 9 quantiles [(τ): 0.1 to 0.9, every 0.1] – (QRF 0.1, …, QRF 0.9), and Random Forest – (RF).

**Table S2.** Mean Square Error for Yield, Total number of fruits, Leaf miner infestation and Cercosporiosis incidence measured in 195 individuals of arabica coffee, using a holdout validation scheme repeated 10 times for GBLUP and all the fitted models used as meta learners.

| Methods | Traits | | | |
| --- | --- | --- | --- | --- |
|  | YL | NF | LM | CER |
| GBLUP | 16.05 (1.01) | 2329.00 (368.99) | 0.21 (0.02) | 0.57 (0.07) |
| SGB2K | 20.39 (2.29) | 2492.53 (265.91) | 0.24 (0.02) | 0.72 (0.1) |
| SM | 16.26 (1.14) | 2133.54 (306.05) | 0.21 (0.02) | 0.55 (0.07) |
| SWR | 22.21 (2.08) | 2455.98 (303.08) | 0.29 (0.03) | 0.78 (0.09) |
| SRNW | 19.81 (2.18) | 2263.16 (238.49) | 0.23 (0.02) | 0.83 (0.11) |
| SRidgeR | 61.56 (6.96) | 7230.96 (808.41) | 2.89 (0.36) | 1.63 (0.19) |
| S_G2Kbest | 16.03 (1.87) | 2371.94 (286.74) | 0.22 (0.02) | 0.61 (0.08) |
| SMBest | 52.38 (3.37) | 8534.97 (1109.36) | 0.49 (0.05) | 0.94 (0.08) |
| SWRBest | 25.27 (2.98) | 3478.61 (536.68) | 0.21 (0.02) | 0.64 (0.06) |
| SRNWBest | 17.82 (2.19) | 2399.84 (293.04) | 0.28 (0.02) | 0.64 (0.09) |
| SRidgeBest | 69.28 (9.18) | 8390.68 (518.50) | 6.65 (0.80) | 3.45 (0.37) |
| RFBest | 24.75 (2.06) | 5083.33 (631.78) | 0.35 (0.02) | 0.54 (0.05) |

SGBLUP - Stacking Genomic Best Linear Unbiased Predictor; S_GBLUPbest - Stacking Ensemble Genomic Best Linear Unbiased Predictor Best; SM - Simple Mean; SMBest – Simple Mean Best; SWR - Stacking Weighed Regression; SWRBest - Stacking Weighed Regression Best; SRNW - Stacking Regression Non-Weighed; SRNWBest - Stacking Regression Non-Weighed Best; SRidgeR – Stacking Ridge Regression; SRidgeBest – Stacking Ridge Regression Best; RFBest – Random Forest Best. The models named as best used in the fitting only the results provided by those methods that presented predictive ability higher the mean
